# Effects of Cannabidiol and Delta-9-Tetrahydrocannabinol on Plasma Endocannabinoid Levels in Healthy Volunteers: A Randomized Double-Blind Four-Arm Crossover Study

**DOI:** 10.1089/can.2022.0174

**Published:** 2024-02-12

**Authors:** Lucy A. Chester, Amir Englund, Edward Chesney, Dominic Oliver, Jack Wilson, Simina Sovi, Alex M. Dickens, Matej Oresic, Tuomas Linderman, John Hodsoll, Amedeo Minichino, John Strang, Robin M. Murray, Tom P. Freeman, Philip McGuire

**Affiliations:** ^1^ Department of Psychosis Studies and Institute of Psychiatry, Psychology and Neuroscience, King's College London, London, United Kingdom.; ^2^ National Addiction Centre (NAC), Institute of Psychiatry, Psychology and Neuroscience, King's College London, London, United Kingdom.; ^3^ Department of Psychiatry, Oxford University, Warneford Hospital, Oxford, United Kingdom.; ^4^ The Matilda Centre for Research in Mental Health and Substance Use, The University of Sydney, New South Wales, Australia.; ^5^ Turku Bioscience Center, University of Turku and Åbo Akademi University, Turku, Finland.; ^6^ Department of Chemistry, University of Turku, Turku, Finland.; ^7^ School of Medical Sciences, Örebro University, Örebro, Sweden.; ^8^ Department of Biostatistics and Health Informatics, Institute of Psychiatry, Psychology and Neuroscience, King's College London, London, United Kingdom.; ^9^ Department of Psychology, University of Bath, Bath, United Kingdom.

**Keywords:** THC, CBD, endocannabinoids, anandamide, 2-arachidonoylglycerol, cannabis

## Abstract

**Background:**

The effects of cannabis are thought to be mediated by interactions between its constituents and the endocannabinoid system. Delta-9-tetrahydrocannabinol (THC) binds to central cannabinoid receptors, while cannabidiol (CBD) may influence endocannabinoid function without directly acting on cannabinoid receptors. We examined the effects of THC coadministered with different doses of CBD on plasma levels of endocannabinoids in healthy volunteers.

**Methods:**

In a randomized, double-blind, four-arm crossover study, healthy volunteers (*n*=46) inhaled cannabis vapor containing 10 mg THC plus either 0, 10, 20, or 30 mg CBD, in four experimental sessions. The median time between sessions was 14 days (IQR=20). Blood samples were taken precannabis inhalation and at 0-, 5-, 15-, and 90-min postinhalation. Plasma concentrations of THC, CBD, anandamide, 2-arachidonoylglycerol (2-AG), and related noncannabinoid lipids were measured using liquid chromatography-mass spectrometry.

**Results:**

Administration of cannabis induced acute increases in plasma concentrations of anandamide (+18.0%, 0.042 ng/mL [95%CI: 0.023–0.062]), and the noncannabinoid ethanolamides, docosatetraenylethanolamide (DEA; +35.8%, 0.012 ng/mL [95%CI: 0.008–0.016]), oleoylethanolamide (+16.1%, 0.184 ng/mL [95%CI: 0.076–0.293]), and N-arachidonoyl-L-serine (+25.1%, 0.011 ng/mL [95%CI: 0.004–0.017]) (*p*<0.05). CBD had no significant effect on the plasma concentration of anandamide, 2-AG or related noncannabinoid lipids at any of three doses used. Over the four sessions, there were progressive decreases in the preinhalation concentrations of anandamide and DEA, from 0.254 ng/mL [95%CI: 0.223–0.286] to 0.194 ng/mL [95%CI: 0.163–0.226], and from 0.039 ng/mL [95%CI: 0.032–0.045] to 0.027 ng/mL [95%CI: 0.020–0.034] (*p*<0.05), respectively.

**Discussion:**

THC induced acute increases in plasma levels of anandamide and noncannabinoid ethanolamides, but there was no evidence that these effects were influenced by the coadministration of CBD. It is possible that such effects may be evident with higher doses of CBD or after chronic administration. The progressive reduction in pretreatment anandamide and DEA levels across sessions may be related to repeated exposure to THC or participants becoming less anxious about the testing procedure and requires further investigation.

The study was registered on clinicaltrials.gov (NCT 05170217).

## Introduction

Cannabis is the world's most used illicit drug,^1^ and regular use is associated with adverse effects on mental health and cognition.^2–6^ On the contrary, one of its constituents, cannabidiol (CBD), is a novel candidate treatment in psychiatry.^7–10^

The main psychoactive component of cannabis, delta-9-tetrahydrocannabinol (THC), is a partial agonist at G-protein–coupled cannabinoid receptors type-1 and type-2 (CB_1_ and CB_2_).^11,12^ THC is responsible for the intoxicating and pleasurable effects of cannabis as well as its adverse effects. CBD is the second most abundant phytocannabinoid in cannabis, and has relatively low affinity for the orthosteric binding sites of CB_1_ and CB_2_.^13^ The endogenous ligands for these receptors are endocannabinoids such as anandamide (AEA) and 2-arachidonoylglycerol (2-AG).^12,14^ Both AEA and 2-AG are high-affinity CB_1_ receptor agonists, while AEA has lower affinity for CB_2_.^15^ The endocannabinoid system (ECS) has been implicated in the regulation of brain development, synaptic plasticity, and neuronal signaling.^14,16,17^

The mechanism by which CBD exerts its effects is unclear. In preclinical studies, CBD can act as a negative allosteric modulator at the CB_1_ receptor, but it does not alter the subjective effects of cannabis associated with THC binding to CB_1_ receptors.^13,18–21^ One hypothesis is that CBD inhibits AEA metabolism, leading to an upregulation in AEA signaling.^22^ *In vitro* experimentation has shown that CBD can reduce AEA degradation by inhibiting both its cellular reuptake through the anandamide membrane transporter and its hydrolysis by the intracellular enzyme fatty acid amide hydrolase.^23^

Other putative mechanisms of action of CBD include inhibiting the metabolism and/or inducing the synthesis of N-acylethanolamines (NAEs).^24,25^ Members of the NAE family include AEA, docosatetraenylethanolamide (DEA), oleoylethanolamide (OEA), and stearoylethanolamide (SEA). Although nonendocannabinoid NAEs such as DEA, OEA, and SEA either do not or weakly exert direct action through CB_1_ or CB_2_, they do have endocannabinoid-like properties.^26,27^

Acute intravenous administration of THC has been shown to transiently increase plasma levels of AEA and 2-AG, through unclear mechanisms.^28^ In contrast, cross-sectional studies suggest that chronic cannabis use can downregulate AEA and possibly upregulate 2-AG signaling.^29–31^ However, the acute dose effects of inhaled THC and CBD in quantities naturally present in cannabis on circulating endocannabinoids have yet to be established.

The aim of this study was to examine the effects of THC and CBD on plasma endocannabinoid levels and related noncannabinoid lipids. Four preparations of cannabis were used, each containing a fixed dose of THC, but a different dose of CBD. We hypothesized that (i) administration of THC would lead to a transient increase in plasma AEA and 2-AG, and (ii) these effects would be modulated by coadministered CBD in a dose-dependent manner.

## Materials and Methods

### Study design

In a randomized, double-blind, four-arm crossover study, healthy volunteers were studied on four occasions. In each session, they received a dose of cannabis vapor containing 10 mg THC plus CBD at a dose of either 0, 10, 20, or 30 mg. These doses were designed to reflect the doses of THC and CBD typically found in recreational cannabis.^32^

### Ethics

The study was approved by the KCL Research Ethics Committee (RESCMR-16/17-4163). Written informed consent was obtained from each participant. The study was conducted in compliance with the principles of Good Clinical Practice and the Declaration of Helsinki (1996), and registered on Open Science Framework (https://osf.io/kt3f7) and clinicaltrials.gov (NCT05170217).

### Study drugs

Raw cannabis plant material was provided by Bedrocan BV, Netherlands. Bedrocan (batch release specifications: 0.1% CBD, 22.6% THC), Bedrolite (7.5% CBD, 0.3% THC), and placebo (<0.1% cannabinoids) were prepared to administer CBD:THC in four different ratios: 0:1, 1:1, 2:1, and 3:1. In all four preparations, the dose of THC was 10 mg (two standard THC units),^33^ whereas the dose of CBD was 0 mg (0:1), 10 mg (1:1), 20 mg (2:1), and 30 mg (3:1), respectively. Placebo cannabis was used to equalize the weight of each preparation (Table 1).

**Table 1.** Depiction of Cannabis Preparations

**CBD:THC ratio**	**0:1**	**1:1**	**2:1**	**3:1**
THC dose (mg)	10	10	10	10
CBD dose (mg)	0	10	20	30
Bedrocan cannabis (mg)	44.2	42.5	40.7	38.9
Bedrolite cannabis (mg)	0.0	132.8	266.1	399.5
Placebo cannabis (mg)	394.2	263.1	131.6	0.0

Batch specifications of cannabis products: Bedrocan: 22.6% THC, 0.1% CBD; Bedrolite: 0.3% THC, 7.5% CBD; placebo: <0.1% THC, <0.1% CBD.

CBD, cannabidiol; THC, delta-9-tetrahydrocannabinol.

### Participants

Participants were 21–50 years of age, had used cannabis at least once previously, had used cannabis < once weekly on average over the last 12 months, were not taking medications (excluding contraceptives), and had no psychiatric or medical history. Details of recruitment and full inclusion/exclusion criteria are listed in the Supplementary Data (p. 2).

### Procedure

The study was conducted at the NIHR Wellcome Trust Clinical Research Facility at King's College Hospital. Each participant attended a screening visit at which a physical and mental health examination and assessment for study eligibility were undertaken by a physician. Participants also practiced the vapor inhalation technique with an air-filled balloon.

### Experimental visits

Each participant attended four experimental visits, with a minimum 7-day washout between visits. Participants were asked to abstain from illicit drugs for the duration of the study, and from alcohol, tobacco, and vaping 24 h before each visit, verified by a urine drug screen, alcohol breath test (BAC=0), and carbon monoxide breath test (CO <10 ppm). Experiments began at either 10:00 or 12:00. An intravenous cannula was inserted, and the baseline blood sample was drawn 30 min (95%CI: 29–33) before drug administration.

The order that participants received the four cannabis preparations (CBD:THC ratios) was randomized. Drug was administered by inhalation using a Volcano Medic Vaporizer (Storz & Bickel, Germany), following the protocol from Lawn et al.^21^ Cannabis was vaporized at 210°C into a covered polythene balloon with a valve mouthpiece, which prevented loss of cannabinoids between inhalations. The same balloon was filled twice using the same cannabis to ensure the full dose was administered. A standardized inhalation procedure was repeated until both balloons had been emptied. During the study visit, participants also completed cognitive and psychological assessments^34^; see Supplementary Data (p. 4).

### Blood collection and analysis

Venous blood samples were collected into lithium-heparin tubes 30 min precannabis inhalation, immediately after the final inhalation (0-min), and at 5-, 15-, and 90-min postinhalation. Samples were centrifuged at 4°C, divided into two cryovials, stored at −20°C until all samples from that day had been collected, then moved to a −80°C freezer.

Plasma concentrations of CBD and THC were determined using high-performance liquid chromatography–mass spectrometry at the Mass Spectrometry Facility, KCL.^35^

Plasma concentrations of AEA and 2-AG, their precursor arachidonic acid (AA), and six biologically related endogenous fatty acid ethanolamides: N-arachidonoyl-L-serine (ARA-S), DEA, OEA, SEA, alpha-linolenoylethanolamide (aLEA), and gamma-linolenoylethanolamide (gLEA) (eFigure 1) were quantified using a validated ultrahigh pressure liquid chromatography-mass spectrometry method (Dickens et al^36^) at the Turku Metabolomics Centre (Turku Bioscience, Finland). As it was not possible to separate 1-AG and 2-AG in plasma due to rapid isomerization,^37^ the quantity was reported as total AG (henceforth described as “2-AG”).

**FIG. 1.**
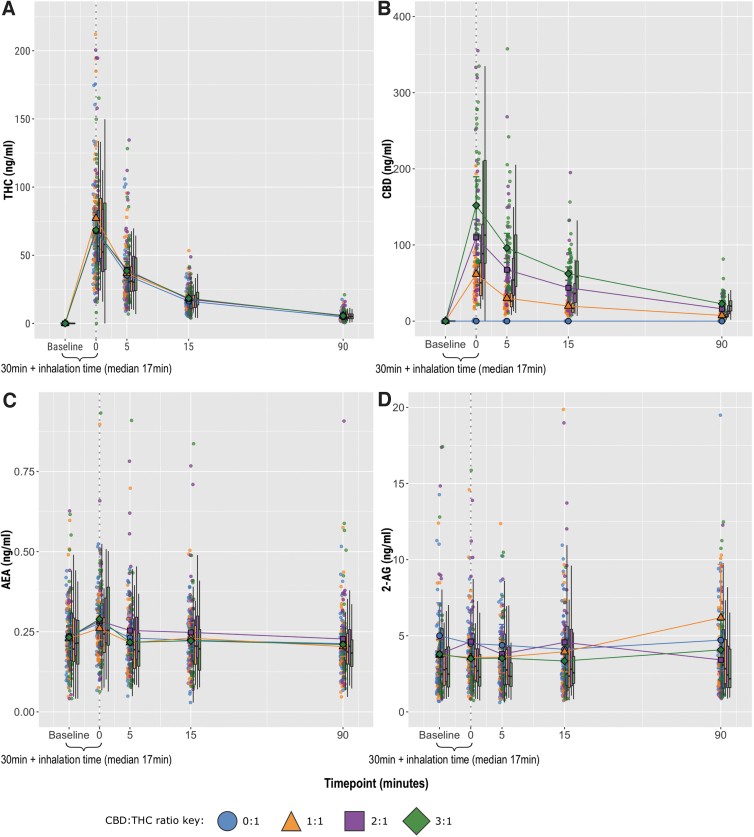
Plasma concentration–time graphs, stratified by CBD:THC ratio. **(A)** THC, **(B)** CBD, **(C)** AEA, **(D)** 2-AG reported as total AG. Circles show individual data points, larger shapes show mean values, and boxplots show median and interquartile range. 2-AG, 2-arachidonoylglycerol; AEA, anandamide; CBD, cannabidiol; THC, delta-9-tetrahydrocannabinol.

### Statistical analysis

All analyses were completed using R, version 3.3.2.^38^ Missing values were imputed using multiple imputation chain equations (MICEs; mice package version 3.13.0)^39^ after confirming no detectable deviation from missing completely at random (MCAR) based on Little's MCAR test. All analyses were completed using linear mixed models (lme4 package version 1.1-26).^40^

The primary outcome of the effects of different CBD:THC ratios on plasma analyte level was measured as peak effects (Model 1) and area under the curve (AUC; Model 2) of mean plasma concentrations. Peak effects (i.e., estimated *C*_max_) were determined as the plasma concentrations at the timepoint at which they were at the highest (estimated *T*_max_). AUC values were calculated after baseline correction using the spline method (DescTools package).^41^ The CBD:THC ratios (0:1, 1:1, 2:1, 3:1) were coded as a categorical variable.

Participant ID was coded as a categorical variable and included as a random effect to account for dependency between repeated measures. Estimated marginal mean (EMM; emmeans package version 1.5.2-1)^42^ differences were calculated for all six contrasts (0:1 vs. 1:1, 0:1 vs. 2:1, etc.). Models 1 and 2 were fully adjusted by including preinhalation plasma concentration (continuous variable) and visit number (categorical variable; visits 1, 2, 3, 4), to account for within-subject differences, as well as the number of days between each of the four experimental visits (continuous variable) to account for the possible carryover effect of repeated exposure to THC.^30,31^ For time between experimental visits, one outlier value was identified using Rosner's generalized extreme Studentized deviate test (GEST; EnvStats package version 2.7.0)^43^ and excluded.

The secondary outcome of the effects of THC on plasma analyte levels was assessed by Model 3. The effect of THC alone was determined by analyzing plasma levels after administration of THC only (0:1 CBD:THC ratio), excluding all other visits (Model 3a). Mean plasma concentrations at each of the timepoints (categorical variable; preinhalation, 0, 5, 15, and 90 min) were compared, including participant ID as a random effect. EMM differences were calculated for all 10 contrasts (preinhalation vs. 0 min, etc.). The fully adjusted Model 3a included the visit number and time since last visit variables. To maximize statistical power, the analysis was then repeated to include all experimental visits (Model 3b). The fully adjusted Model 3b included the CBD:THC ratio, visit number, and time since last visit variables.

Exploratory analyses assessed changes in plasma analyte levels over the experimental visits (Model 4). Model 4a compared preinhalation concentrations of the analytes between the four visits, with participant ID as a random effect. EMM differences were calculated for all six contrasts (visit 1 vs. visit 2, etc.). In *post hoc* analyses, we assessed whether any identified effects were influenced by CBD. Preinhalation levels of analytes at visits 2, 3, and 4 (Models 4b–d, respectively) were compared with total CBD dose from previous visits (categorical variable). Models 4a–d were fully adjusted by including the time since last experimental visit variable.

*Post hoc* analyses to explore sex differences in endocannabinoid responses to THC and/or CBD were performed by adding sex (categorical variable) as an interaction term to the predictor variable in each model.

EMM differences were corrected for multiple comparisons using the Tukey adjustment method, and are presented along with *p*-values and 95% confidence intervals.

## Results

Sixty-four potential participants were randomized, of whom 46 completed all four experimental sessions and contributed data. Demographics and physical characteristics are shown in Table 2. Median inhalation time was 17 min (IQR=11). The median time between experimental visits was 14 days (IQR=20). For results of cognitive and psychological assessments see Englund et al.^34^

**Table 2.** Demographics of Participants at Baseline

**Variables**	***N* (%)**	**Mean (SD)**
Gender		
Male	25 (54.3)	
Female	21 (45.7)	
Age		26.62 (4.94)
Ethnicity		
White	21 (45.7)	
Asian	10 (21.7)	
Mixed	3 (6.5)	
Black	1 (2.2)	
Other	11 (23.9)	
BMI (kg/m^2^)		23.72 (2.57)
Body fat (%), male		15.56 (5.50)
Body fat (%), female		25.50 (6.33)
Days since last use of alcohol		4.17 (4.62)
Alcohol use/month (days)		8.02 (4.86)
eCigarette use (ever)	12 (26.1)	
Daily eCigarette user	1 (2.2)	
Tobacco use (ever; separate from cannabis)	34 (73.9)	
Daily tobacco user (separate from cannabis)	3 (6.5)	
Use tobacco with cannabis	36 (78.3)	
Age of first cannabis use		17.67 (2.46)
Years of cannabis use		6.63 (4.68)
Cannabis use/year		8.91 (12.67)

### Plasma CBD and THC concentrations

Figure 1 shows the mean plasma concentrations of the endocannabinoids, plus CBD and THC for comparison, versus time, stratified by CBD:THC ratio. The peak and AUC THC concentration remained similar across the four conditions (*p*>0.05), and there was a dose-dependent increase in peak and AUC plasma CBD as the CBD:THC ratio increased (*p*<0.001, eTable 1).

### Comparison of CBD:THC ratios

**FIG. 2.**
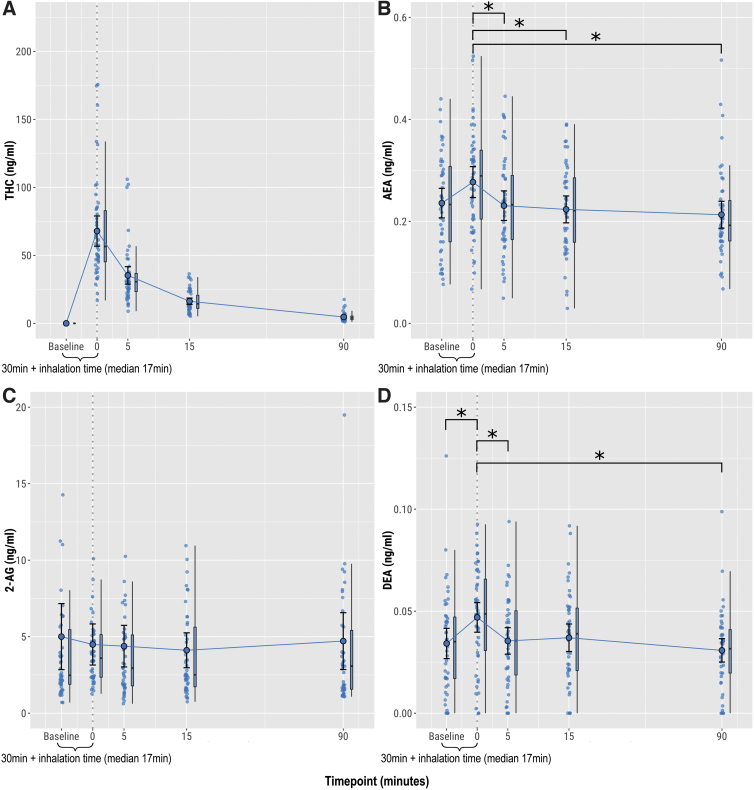
Plasma concentrations after administration of 10 mg THC, 0 mg CBD (0:1 ratio). **(A)** THC, **(B)** AEA, **(C)** 2-AG reported as total AG, **(D)** DEA. Circles show individual data points, larger circles show mean values, and boxplots show median and interquartile range. *p<0.05. DEA, docosatetraenylethanolamide.

There were no significant differences in either peak or AUC plasma concentrations for any of the endocannabinoids or related noncannabinoid lipids between CBD:THC ratios (Fig. 1, e Figure 2, and e Table 1). The estimated *T*_max_ was 0 min for AEA, aLEA, ARA-S, DEA, OEA, and SEA; 5 min for AA and gLEA; and 90 min for 2-AG. For gLEA, the lowest plasma level was selected since levels decreased postinhalation.

### Effect of drug administration

#### THC alone

**FIG. 3.**
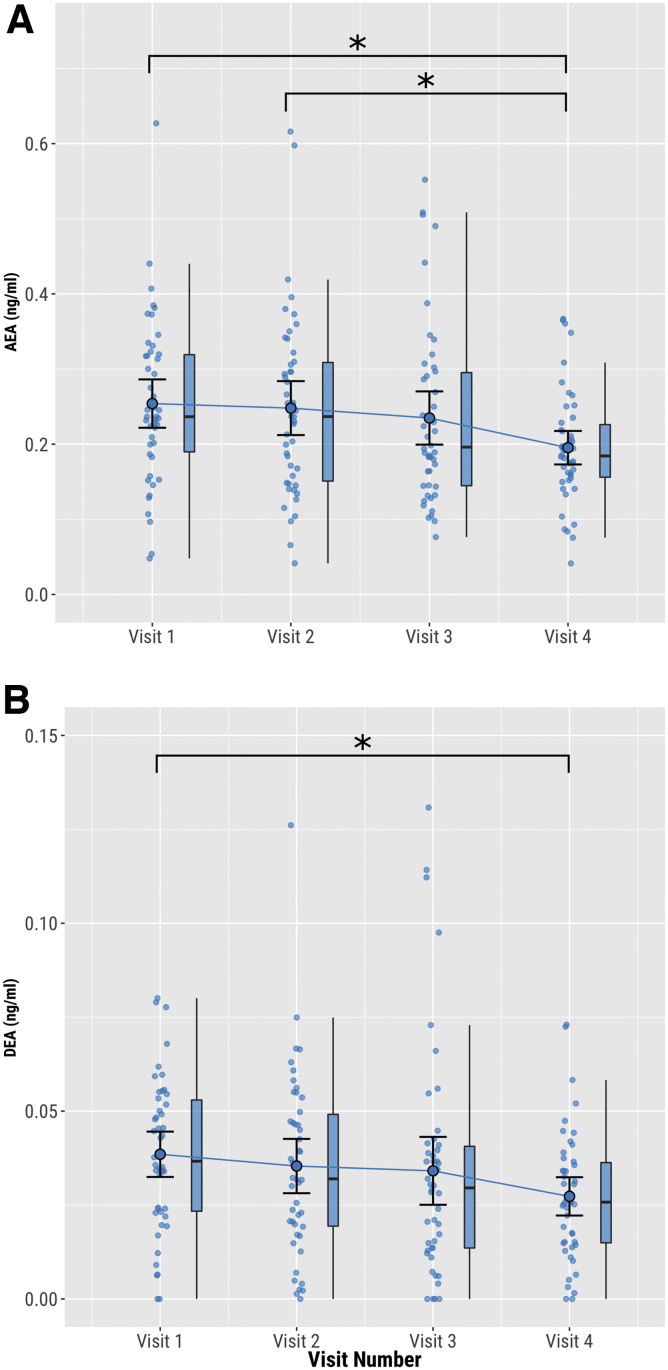
Preinhalation plasma concentrations vs. visit number. **(A)** AEA, **(B)** DEA. Circles show individual data points, larger circles show mean values, and boxplots show median and interquartile range. *p<0.05.

When limiting data to the visits where cannabis containing only THC was administered (0:1 CBD:THC ratio), mean DEA concentration rose by 37.8% (0.013 ng/mL [95%CI: 0.005–0.020], *t*(180)=3.273, *p*=0.011) at 0 min postinhalation, before falling to preinhalation levels by 5 min (Fig. 2). While the mean AEA concentration was greater at 0 min than at 5, 15, or 90 min (*p*<0.05), it was not significantly higher than preinhalation (+17.0%, 0.040 ng/mL [95%CI: 0.010–0.070], *t*(180)=2.633, *p*=0.069) (Fig. 2). There were no significant changes in plasma levels of any of the other endocannabinoids or related noncannabinoid lipids (eTable 2 and e Figure 3).

#### Overall effect of THC

The above analysis was extended to include all experimental visits (i.e., including those in which THC was coadministered with CBD). Plasma levels of AEA, DEA, OEA, and ARA-S increased significantly postcannabis inhalation (eFigure 4). Mean AEA concentration rose by 18.0% (0.042 ng/mL [95%CI: 0.023–0.062], *t*(858)=4.298, *p*<0.001); mean DEA concentration rose by 35.8% (0.012 ng/mL [95%CI: 0.008–0.016], *t*(858)=5.797, *p*<0.0001); mean OEA concentration rose by 16.1% (0.184 ng/mL [95%CI: 0.076–0.293], *t*(858)=3.332, *p*=0.008); and mean ARA-S concentration increased by 25.1% (0.011 ng/mL [95%CI: 0.004–0.017], *t*(858)=3.326, *p*=0.008) immediately postinhalation, before falling to preinhalation levels by 5 min. There were no significant changes in plasma levels of any of the other analytes (eTable 3).

### Effect of visit order on endocannabinoid levels

Between visit 1 and visit 4, the mean preinhalation AEA concentration fell by 23.6% (0.060 ng/mL [95%CI: 0.024–0.096], *t*(135)=3.278, *p*=0.007), and the mean preinhalation DEA concentration fell by 29.1% (0.011 ng/mL [95%CI: 0.003–0.019], *t*(135)=2.779, *p*=0.031) (Fig. 3). After adjusting for time between visits, the decrease in baseline DEA no longer reached statistical significance (*p*=0.086) (eTable 4). *Post hoc* analyses showed that none of preinhalation concentrations of AEA and DEA at visits 2, 3, and 4 were associated with the total dose of CBD received at the previous visits (*p*>0.05) (eTable 5). There were no significant changes in preinhalation plasma levels of any of the other analytes across experimental visits (eTable 4).

### Sex differences

There were no significant sex differences in the endocannabinoid or related noncannabinoid lipid responses to THC or CBD, with the exception of Models 3b and 4a for SEA. However, these results were found to be caused by two outliers, identified using Rosner's generalized extreme Studentized deviate test, and were no longer significant when these outliers were removed; see Supplementary Data (p. 50).

## Discussion

To our knowledge, this is the first study to investigate the acute effects of coadministered THC and CBD on plasma endocannabinoid concentrations. Its strengths include the use of a double-blind, within-subjects design, which mitigated against potential placebo effects related to CBD, as well as interindividual differences in response to THC and CBD. Restricting participation to infrequent cannabis users reduced the risk of prior cannabis use impacting circulating endocannabinoid levels.

We did not detect an effect of the CBD:THC ratio in cannabis on the plasma concentration of any of the tested endocannabinoids or related lipid compounds. Previous research has indicated that CBD may enhance AEA signaling. Leweke et al^22^ reported that treatment with 800 mg of oral CBD for 14 days led to an increase in AEA and OEA in patients with psychosis, with AEA serum levels increasing 1 pmol/mL (equivalent to 0.348 ng/mL) after 28 days. However, another study found that 200 mg of CBD daily for 13 weeks had no effect on plasma levels of AEA, 2-AG, or OEA in patients with type 2 diabetes.^44^

The absence of an effect on plasma endocannabinoids in our study may have been due to the administration of single doses of CBD at relatively low dosages. Comparing doses between oral and vaporized CBD is difficult due to the differences in pharmacokinetics between formulations; CBD undergoes significant first-pass metabolism,^45^ and its absorption and elimination are slower when taken orally versus inhalation.^46^

Nevertheless, an oral dose of 800 mg CBD will produce much greater systemic availability of the drug than our maximum inhaled dose of 30 mg CBD.^46^ The doses of THC and CBD that we used were designed to reflect those typically found in recreational cannabis.^32^ As typical “joint” contains between 300–350 mg of cannabis material,^47^ it would not be possible for cannabis used recreationally to provide quantities of CBD equivalent to an 800 mg oral dose.

The inhalation of vaporized cannabis containing 10 mg THC led to transient increases in plasma levels of AEA and the endocannabinoid-like lipids DEA, OEA, and ARA-S. These findings are consistent with those of Thieme et al,^28^ who found that plasma AEA increased by 0.060 ng/mL 30 min after an IV dose of 0.1 mg/kg IV THC. However, we did not detect the increase in plasma 2-AG reported by Thieme et al.^28^

Walter et al^48^ found that 20 mg THC given orally (as dronabinol) produced higher concentrations of AEA, OEA, and 2-AG after 2 and 3 h compared with placebo. In contrast, Kearney-Ramos et al^49^ did not detect any changes in either plasma AEA or 2-AG after the inhalation of an estimated 30 mg THC in 26 near-daily cannabis users. This may be explained by frequent cannabis use leading to compensatory adaptations in the ECS, examples including reductions in circulating endocannabinoids and CB_1_ receptor availability.^50–53^

The increase in AEA, DEA, OEA, and ARA-S plasma concentrations immediately postdrug administration could be due to a direct effect of THC on either their synthesis or degradation. It is also possible that THC indirectly increased endocannabinoid levels through enhanced catecholaminergic and glucocorticoid signaling, which are known to cause significant increases in plasma endocannabinoid concentrations.^54–58^ THC may also have simply displaced the endogenous ligands, which have a similar protein binding profile, particularly ligands of the GPR55 receptor, which include AEA, OEA, and ARA-S.^59–61^

Preinhalation levels of AEA and DEA decreased in a stepwise manner between the first and final experimental visit. Differences in CBD dose between sessions did not alter these results, suggesting that CBD was not a factor. However, repeated doses of THC have been shown to downregulate AEA and 2-AG signaling in the rat striatum.^31^ Similarly in humans, frequent cannabis users have lower cerebrospinal fluid (CSF) concentrations of AEA than infrequent users.^30^

Our results are unlikely to be due to a direct pharmacological action of THC on the synthesis or degradation of AEA, as adjusting the model for time between experimental sessions (minimum 7 days) had no significant impact, and preinhalation plasma samples taken at each visit consistently found no measurable THC or CBD postwashout. Another possible explanation is that as participants became increasingly familiar with the experimental sessions, there may have been a reduction in the stress associated with the procedure. Stress can induce glucocorticoid and catecholamine responses that can increase AEA release.^54,55^ Future studies may wish to explore if the gradual decrease of baseline AEA represents a conditioned response to the experimental setting.

Certain limitations should be considered in the interpretation of the data. CSF levels of AEA are not correlated with those in peripheral blood, so plasma levels of endocannabinoids do not necessarily reflect those present in brain.^62^ The duration of cannabis inhalation varied significantly between participants and between experiments, with a median duration of 17 min. Future studies should consider methods to standardize duration of inhalation. Because the absorption of cannabinoids will have started before the end of the inhalation period, referring to the first timepoint as “0 min” is not strictly accurate.

This also limits our ability to compare the sampling timelines of this study with those of Thieme et al^28^ or Walter et al,^48^ as the routes and durations of administration were different.^45,63^ It is possible that food consumption could have impacted levels of endocannabinoids.^64,65^ Our participants were asked to eat their usual breakfast, but its timing and content were not controlled. The study did not include a placebo THC condition, so we cannot exclude the possibility that the inhalation procedure itself, rather than THC administration, produced changes in AEA, DEA, OEA, and/or ARA-S.

## Conclusions

Inhalation of vaporized cannabis increased levels of plasma AEA and several endocannabinoid-like lipids, but there was no evidence that CBD influenced any of these effects. It is possible that either the doses of CBD were too low to have measurable influence, and/or CBD affected central but not peripheral endocannabinoids. There was a progressive reduction in the plasma concentrations of AEA and DEA across successive experimental sessions, which could reflect a downregulation of endocannabinoid signaling with repeated THC administration, or habituation with the testing procedure.

## Supplementary Material

Supplemental data

## Abbreviations Used

2-AG: 2-arachidonoylglycerol
AA: arachidonic acid
AEA: anandamide
aLEA: alpha-linolenoylethanolamide
ARA-S: N-arachidonoyl-L-serine
AUC: area under the curve
CBD: cannabidiol
CSF: cerebrospinal fluid
DEA: docosatetraenylethanolamide
ECS: endocannabinoid system
EMM: estimated marginal mean
gLEA: gamma-linolenoylethanolamide
MCAR: missing completely at random
NAE: N-acylethanolamine
OEA: oleoylethanolamide
SEA: stearoylethanolamide
THC: delta-9-tetrahydrocannabinol
