# Supplementary material for: Effects of Cannabidiol and Delta-9-Tetrahydrocannabinol on Plasma Endocannabinoid Levels in Healthy Volunteers: A Randomized Double-Blind Four-Arm Crossover Study
Source: Cannabis Cannabinoid Res. 2024 Feb 12;9(1):188–98. doi: 10.1089/can.2022.0174 (PMC10874814; doi:10.1089/can.2022.0174)
Supplement: Supplemental data [file Suppl_Materials.pdf]

Supplementary Materials

Lucy A Chester, Amir Englund, Edward Chesney, Dominic Oliver, Jack Wilson, Simina Sovi, Alex M Dickens, Matej Oresic, Tuomas Linderman, John Hodsoll, Amedeo Minichino, John Strang, Robin M Murray, Tom P Freeman, Philip McGuire. Effects of cannabidiol and delta-9-tetrahydrocannabinol on plasma endocannabinoid levels in healthy volunteers: a randomised double-blind four-arm cross-over study.

Table of Contents

EMETHODS 2

STUDY DRUGS 2

PARTICIPANTS 2

Recruitment 2

Inclusion/Exclusion criteria 2

SCREENING VISIT 3

RANDOMISATION AND MASKING 3

EXPERIMENTAL VISIT 3

EFIGURE 1. TIMELINE OF BASELINE AND EXPERIMENTAL SESSIONS. 4

INHALATION PROCEDURE 5

EFIGURE 2. MOLECULAR STRUCTURE OF PLANT CANNABINOIDS, ENDOCANNABINOIDS AND BIOLOGICALLY RELATED COMPOUNDS ANALYSED FROM PLASMA SAMPLES 6

EFIGURE 3. PLASMA CONCENTRATION-TIME GRAPHS, STRATIFIED BY CBD:THC RATIO 8

EFIGURE 4. PLASMA CONCENTRATIONS FOLLOWING 10MG THC, 0MG CBD (0:1 RATIO) 10

EFIGURE 5. PRE-INHALATION PLASMA CONCENTRATIONS VS. VISIT NUMBER 11

ETABLE 1. PHARMACOKINETICS FOR EACH CONTRAST BETWEEN CBD:THC RATIOS – MODELS 1 AND 2 13

ETABLE 2. PLASMA CONCENTRATIONS VS. TIME FOLLOWING 10MG THC, 0MG CBD (0:1 RATIO) – MODEL 3A 25

ETABLE 3. PLASMA CONCENTRATIONS VS. TIME FOLLOWING 10MG THC (ALL CBD:THC RATIOS) – MODEL 3B 34

ETABLE 4. PRE-INHALATION PLASMA CONCENTRATIONS VS. VISIT NUMBER – MODEL 4A 43

ETABLE 5. PRE-INHALATION PLASMA CONCENTRATIONS OF AEA AND DEA VS. TOTAL CBD DOSE FROM PREVIOUS VISITS – MODELS 4B, 4C AND 4D. 48

ERESULTS 50

SEX DIFFERENCES 50

EFFECT OF PEAK THC CONCENTRATION 50

REFERENCES 51

eMethods

Study Drugs

Standardised cannabis plant material is produced according to Good Manufacturing Practice (GMP) and meet the European Medicines Agency's contaminant levels for products used in the respiratory tract. The products are regulated by the Dutch government's Office of Medicinal Cannabis at the Dutch Ministry of Health, Welfare and Sport.

Participants

Recruitment

Email advertisements were sent to staff and students at King's College London. After completing an initial phone screening, participants were invited to attend a baseline session at King's College Hospital.

Inclusion/Exclusion criteria

Inclusion criteria:

- i) Be aged between 21-50 years old
- ii) Have used cannabis at least once in the past
- iii) Willing and able to provide written informed consent
- iv) Willing to provide blood samples
- v) Be a fluent English speaker

Exclusion criteria:

- i) cannabis use more than one day per week on average over the last 12 months;
- ii) any past or present major mental illness
- iii) a past or present major physical illness
- iv) a score of 5 and above on the Michigan Alcoholism Screening Test [1];
- v) a score of 5 and above on the Fagerstrom Test for Nicotine Dependence [2];
- vi) a score of 5 and above on the Drug Abuse Screening Test (DAST-20) [3];
- vii) past or present use of anti-psychotic or anti-depressant medication;
- viii) a first-degree relative with a psychotic disorder;
- ix) currently use of psychotropic medication;
- x) BMI classified as obese or underweight;
- xi) having taken part in any drug study within the last 30 days or taking part in another study over the course of the trial;
- xii) a known drug sensitivity/allergy towards cannabis or lorazepam; and
- xiii) pregnancy (current or planned) or lactation in women.

Urine pregnancy tests were performed in all female participants at the start of each study visit with drug administration only performed upon a negative result. In addition, participants were asked not to use recreational/illicit drugs at least 7 days and alcohol and tobacco for 24 hours before each visit, confirmed by a urine drug test, alcohol breath test (BAC=0) and carbon monoxide breath test (CO <10 ppm) at each study visit, respectively. Urine drug testing kits tested for amphetamines, benzodiazepines, buprenorphine, cocaine, cannabis, methadone, methamphetamines, morphine, and opiates. Failure to pass tests resulted in rescheduling of the study visit.

Screening Visit

A study physician was present at screening visits to complete a general medical history and brief physical examination, to exclude subjects with medical conditions. Participants were given the opportunity to discuss the patient information sheet and ask questions before completing consent forms. Psychological scales were completed before the participant was discharged.

Randomisation and masking

The study design was a 4 phase cross-over with each phase corresponding to one of the 4 CBD:THC dosing ratios: 0:1, 1:1, 2:1, 3:1. Each participant was required to complete all phases corresponding to all ratios of cannabis preparations. For 4 conditions there are 24 possible permutations of sequential order, e.g., Visit 1, 2:1 – Visit 2, 0:1 – Visit 3, 1:1 – Visit 4, 3:1. Randomised sequences were generated in blocks with the first 24 participants allocated each of the 24 possible orders of ratios, as were the next 24 and so on. Where there were fewer participants than possible order sequences, each participant received a random selection from the 24, sampled without replacement. The randomisation list was generated by a statistician not involved with the study, using a customised randomisation script generated in R software v 3.1 (available on request).

The randomisation was double blinded to both researchers and participants. The randomisation list was passed from the independent statistician to the Maudsley Pharmacy who prepared the cannabis preparations. The pharmacy dispensed the study drug to a blinded researcher. The cannabis preparation was then loaded into the filling chamber and vaporised by a research nurse at the Clinical Research Facility (CRF) who was not involved with any other study procedures. Once filled, this was encased with an opaque bag to ensure the blind was maintained (a higher CBD:THC ratio produces a denser vapour). Upon completion of data collection and entry the randomisation schedule was revealed to the research team prior to data analysis.

Experimental visit

Experimental sessions were scheduled to begin at either 10AM or 12PM. Participants were asked to have a normal (by their own standards) amount of sleep and eat a normal breakfast before arriving. An intravenous cannula was inserted into the participants' arm before the administration of vaporised cannabis.

Rescue medication (oral Lorazepam, 1-2mg prn) was available on request of the participant should they become overly distressed by the effects of the drug but was never required. The participant remained in the CRF until the effects of the drug had worn off (roughly 3 hours after cannabis inhalation). Participants were discharged after completing a field sobriety test and vital signs, and told to avoid driving, operating heavy machinery, or cycling for the next 24 hours. The researchers ensured the participant had contact details to the study physician and knew to contact them with any concern relating to participation in the study or side-effects.

eFigure 1. Timeline of baseline and experimental sessions.

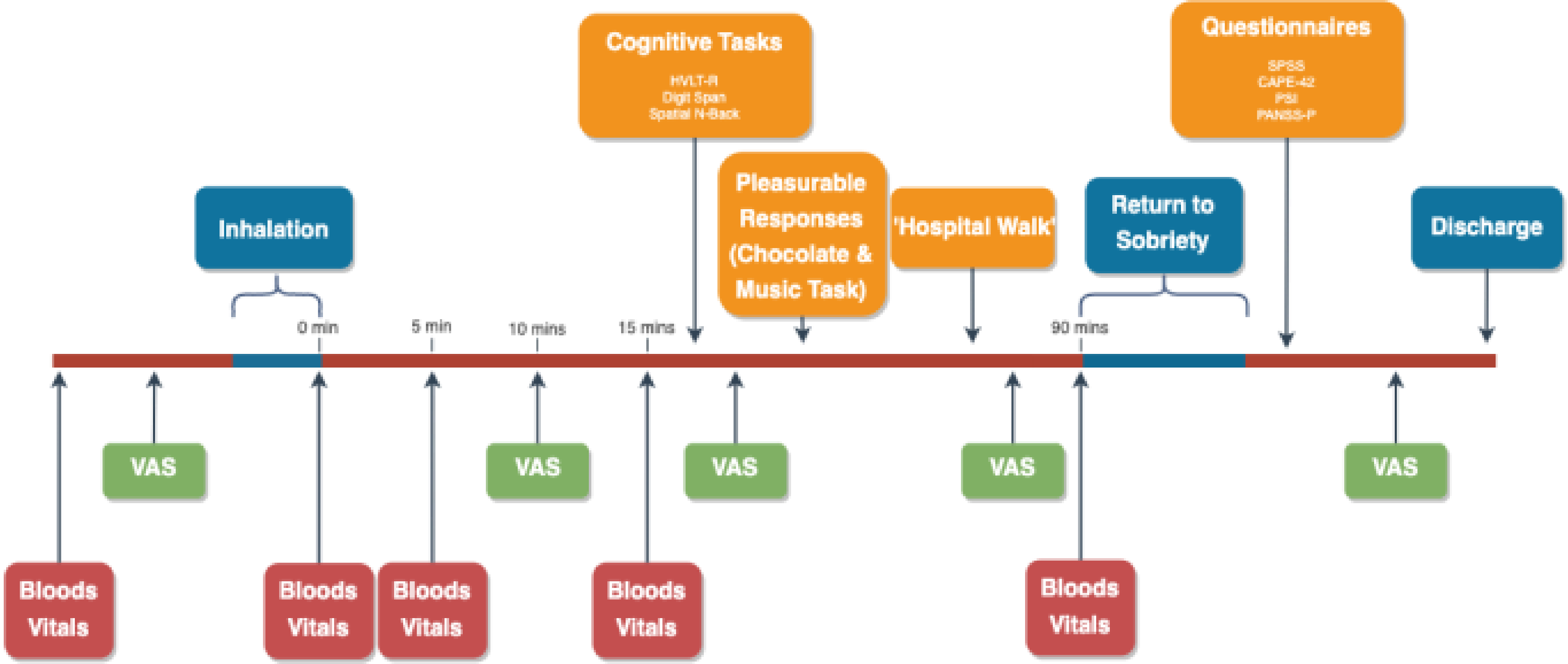

Bloods, blood sample; Vitals, vital signs (HR, BP, O_2 sat.); CAPE-42, Community assessment of Psychic Experiences – state [4]; Digit Span, Forward and reverse digit span; HVLT-R, Hopkins verbal learning task – Revised [5]; PANSS-P, Positive and negative syndrome scale – positive subscale [6]; PSI, Psychotomimetic states inventory [7]; Spatial N-Back task [8]; SPSS, State social paranoia scale [9]; VAS, Visual Analogue Scale.

The timings of the blood draws were based on the known pharmacokinetic profiles of inhaled THC and CBD.[10,11]

See study protocol for full description of timeline and tasks: https://osf.io/kt3f7

Inhalation procedure

Participants were instructed to inhale and hold their breath for 8 seconds before exhaling fully and resting for 8 seconds. Participants inhaled the first balloon standing upright and the second lying on a clinical bed in preparation of blood sampling.

Vaporization of was chosen as the method of drug preparation replicates the bioavailability characteristics of with smoking, while avoiding toxic chemicals from burnt cannabis. A cup of hot lemon and honey water was provided to help with the abrasiveness of cannabis inhalation.

Sample storage and transport

Plasma samples were stored at −80 °C for 329 days (95%CI: 310 to 348) at King's College Hospital, before being transported via a temperature-controlled courier to Turku Metabolomics Centre (Turku Bioscience, Finland).

eFigure 2. Molecular structure of plant cannabinoids, endocannabinoids and biologically related compounds analysed from plasma samples

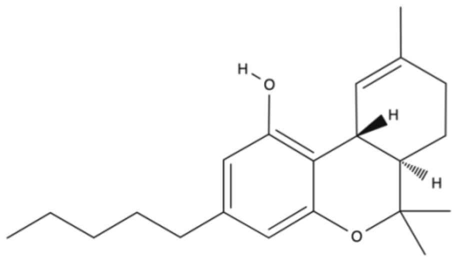

Delta-9-tetrahydrocanabinol - THC

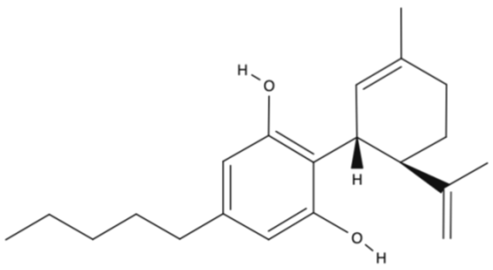

Cannabidiol - CBD

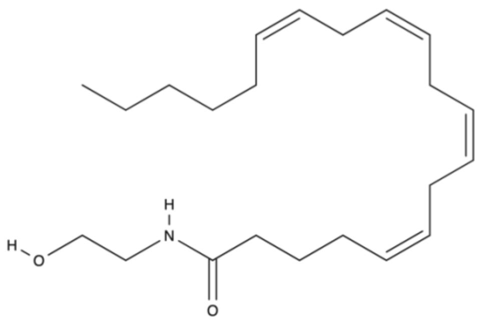

Anandamide - AEA

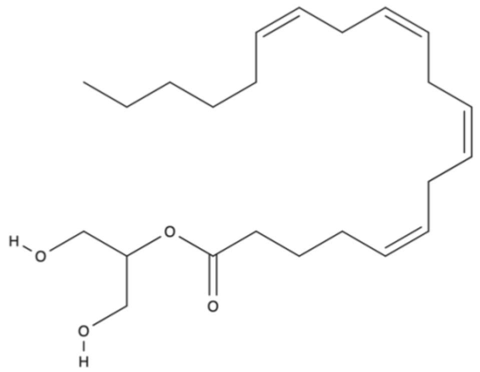

2-Arachidonoylglycerol - 2-AG

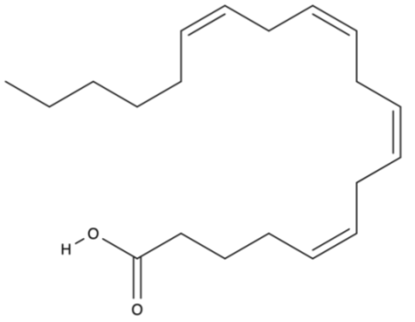

Arachidonic acid - AA

eFigure 2 continued.

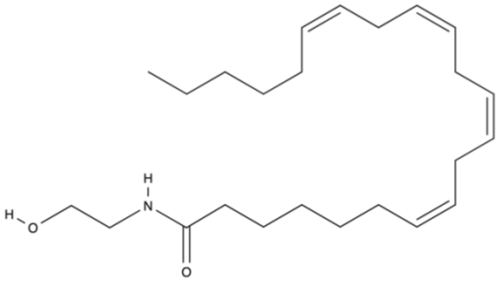

Docosatetraenylethanolamide - DEA

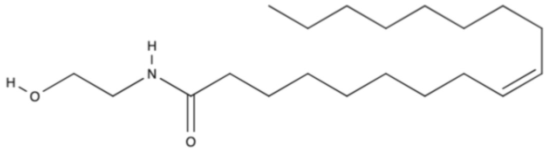

Oleoylethanolamide - OEA

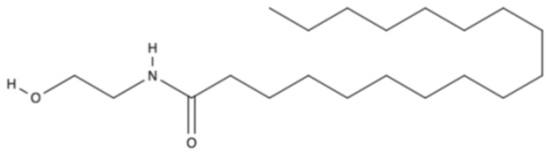

Stearoylethanolamide - SEA

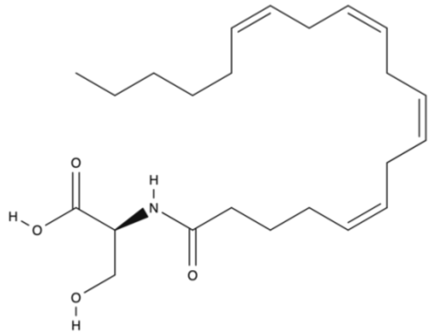

N-arachidonyl-L-serine - ARA-S

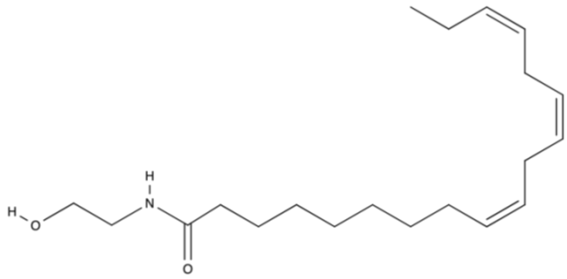

Alpha-linolenoylethanolamide - aLEA

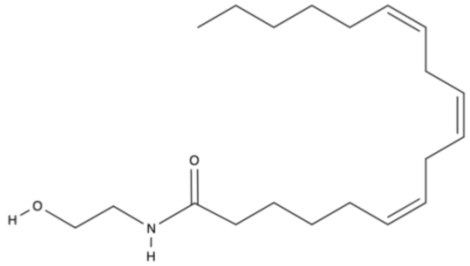

Gamma-linolenoylethanolamide - gLEA

eFigure 3. Plasma concentration-time graphs, stratified by CBD:THC ratio

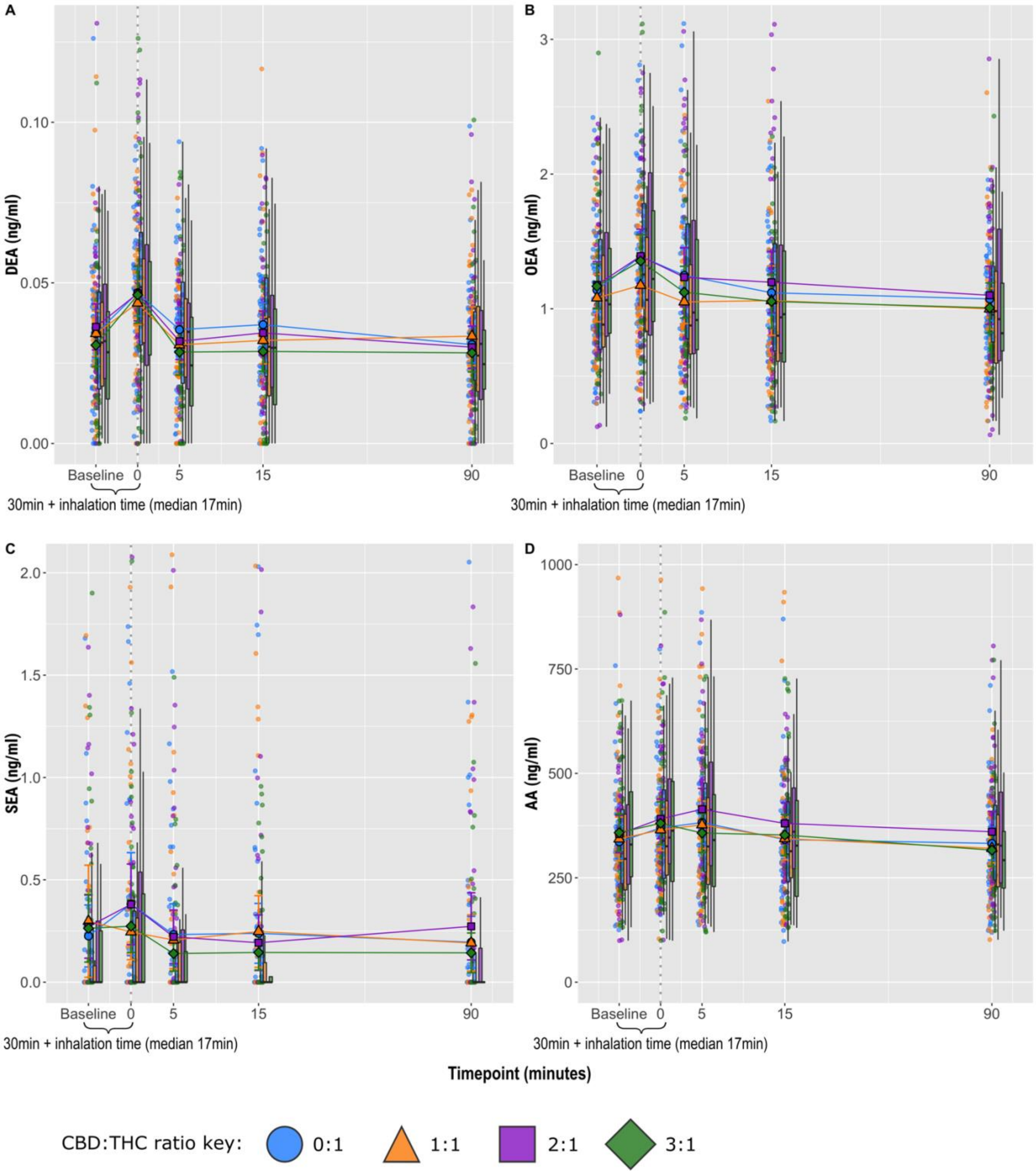

A. Docosatetraenylethanolamide (DEA), **B**. oleoylethanolamide (OEA), **C**. stearoylethanolamide (SEA), **D**. arachidonic acid (AA).

Circles show individual data points, larger shapes show mean values and boxplots show median and interquartile range.

Continued next page.

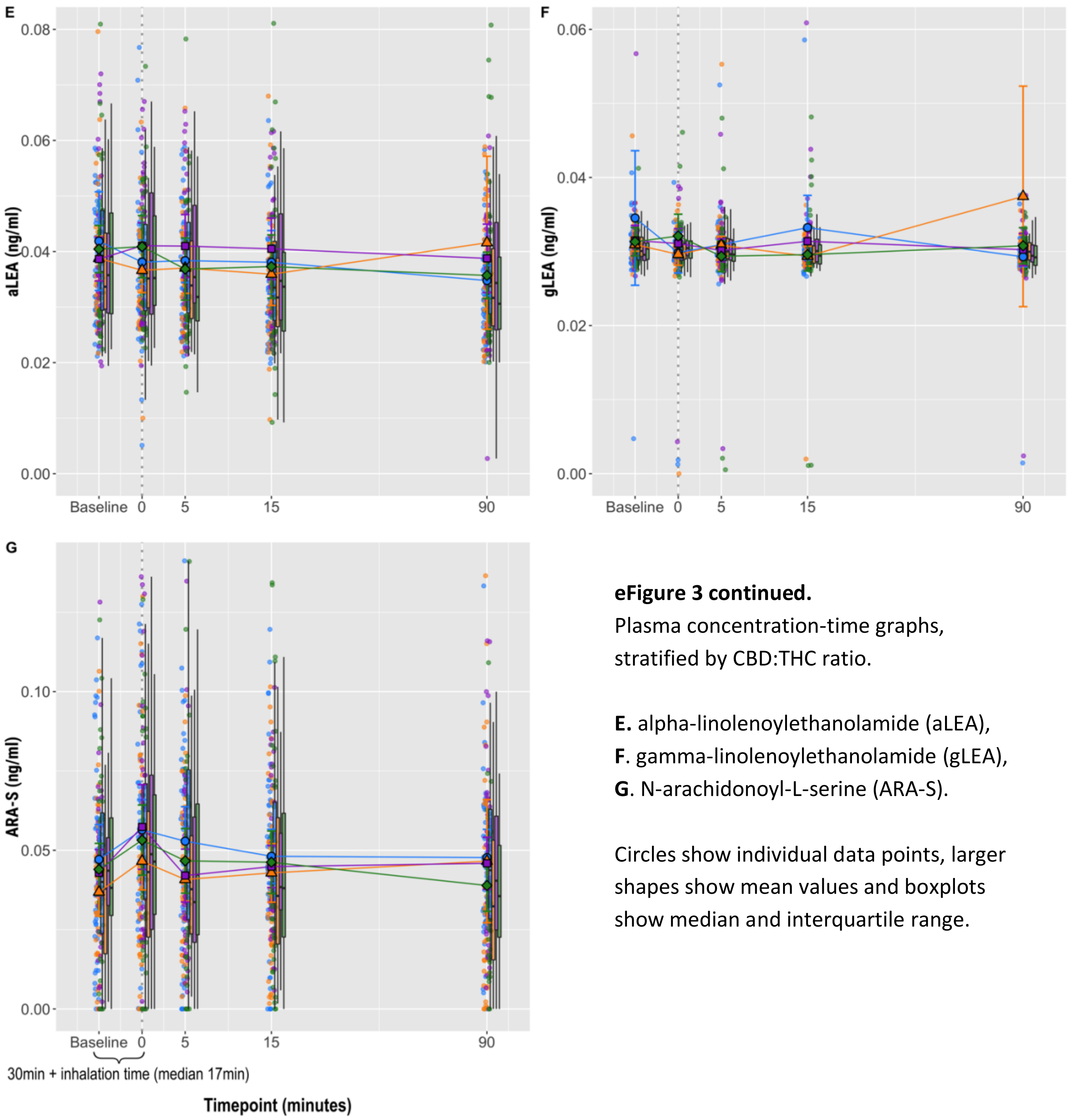

eFigure 3 continued.
Plasma concentration-time graphs, stratified by CBD:THC ratio.

E. alpha-linolenoylethanolamide (aLEA),
F. gamma-linolenoylethanolamide (gLEA),
G. N-arachidonoyl-L-serine (ARA-S).

Circles show individual data points, larger shapes show mean values and boxplots show median and interquartile range.

eFigure 4. Plasma concentrations following 10mg THC, 0mg CBD (0:1 ratio).

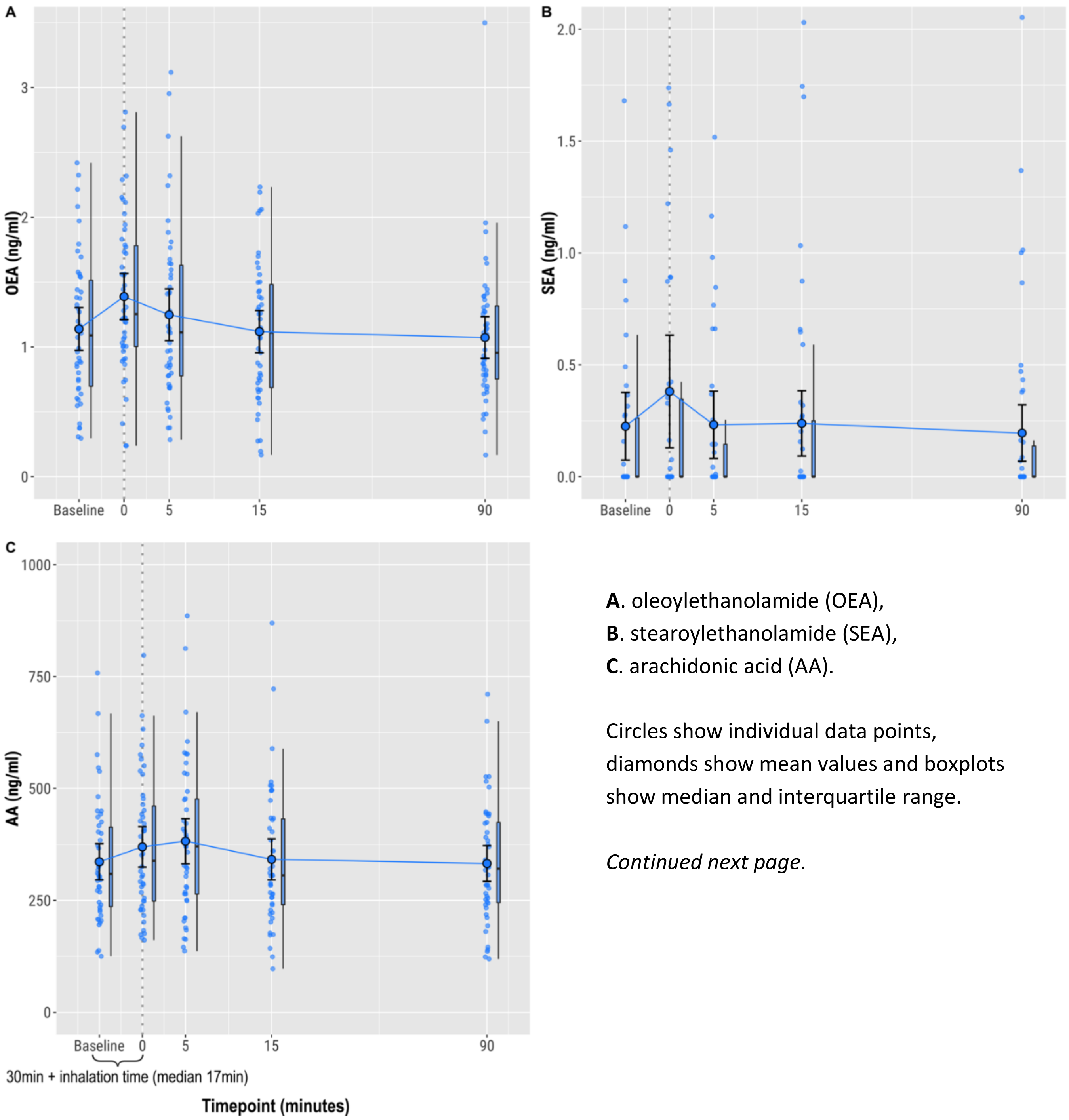

A. oleoylethanolamide (OEA),
B. stearoylethanolamide (SEA),
C. arachidonic acid (AA).

Circles show individual data points, diamonds show mean values and boxplots show median and interquartile range.

Continued next page.

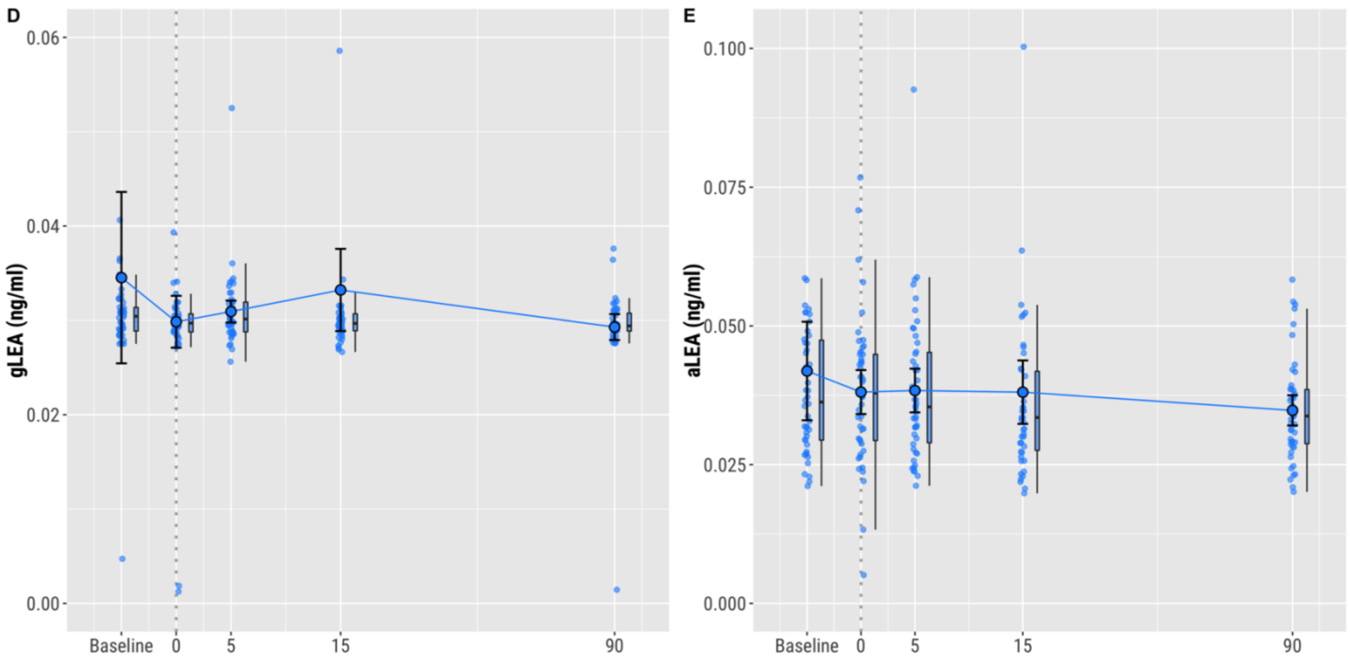

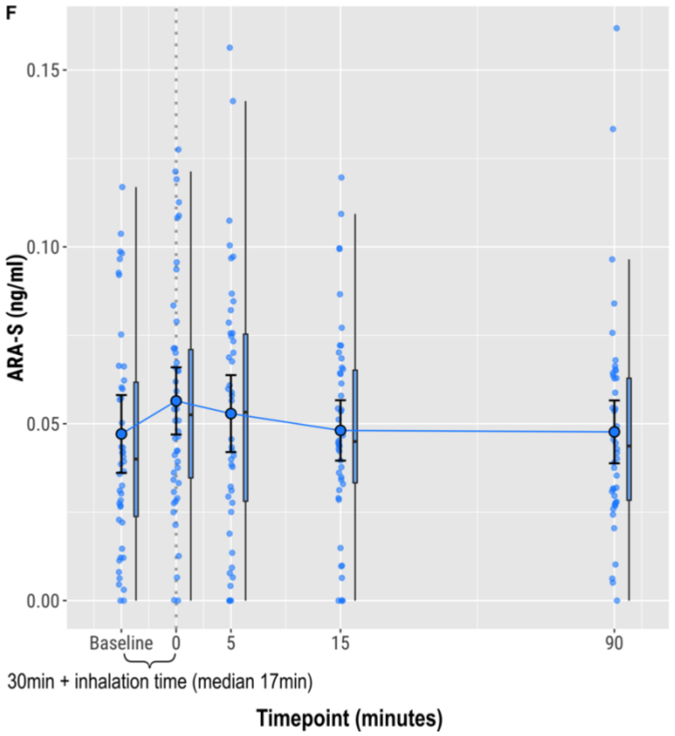

eFigure 4 continued.
Plasma concentrations following administration of 10mg THC, 0mg CBD (0:1 ratio).

E. gamma-linolenoylethanolamide (gLEA),
F. alpha-linolenoylethanolamide (aLEA),
G. N-arachidonoyl-L-serine (ARA-S).

Circles show individual data points, diamonds show mean values and boxplots show median and interquartile range.

eFigure 5. Pre-inhalation plasma concentrations vs. visit number.

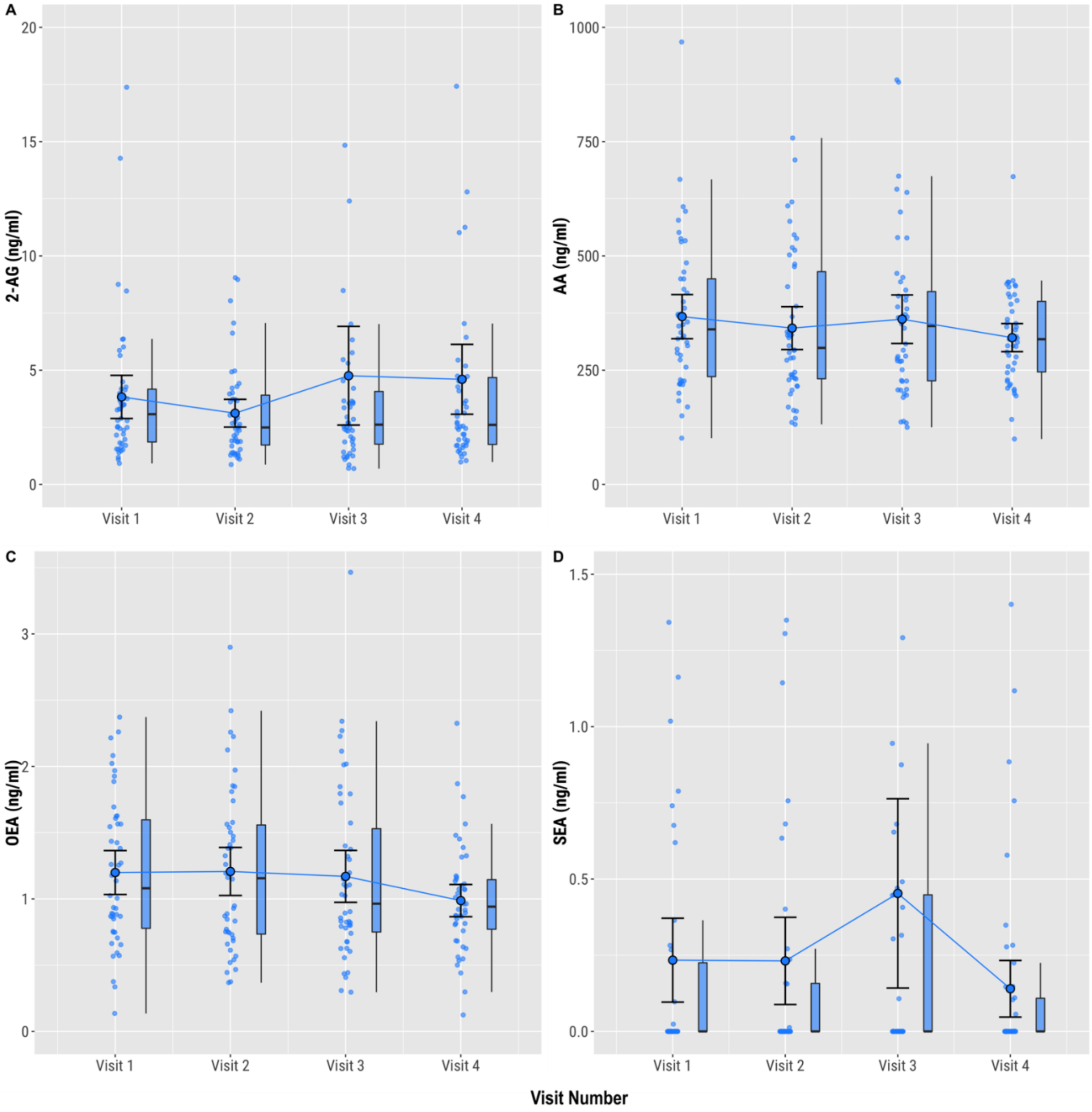

A. 2-arachidonoylglycerol (2-AG), **B**. arachidonic acid (AA), **C**. oleoylethanolamide (OEA), **D**. stearoylethanolamide (SEA).

Circles show individual data points, diamonds show mean values and boxplots show median and interquartile range.

Continued next page.

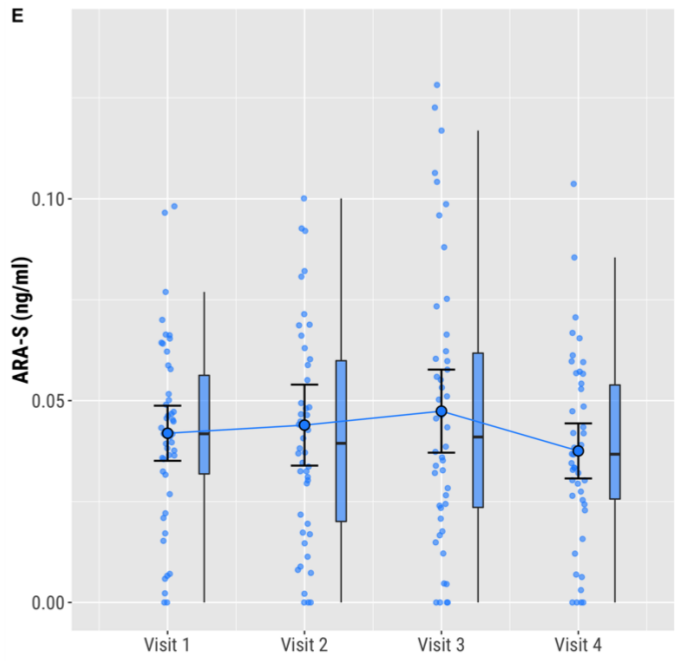

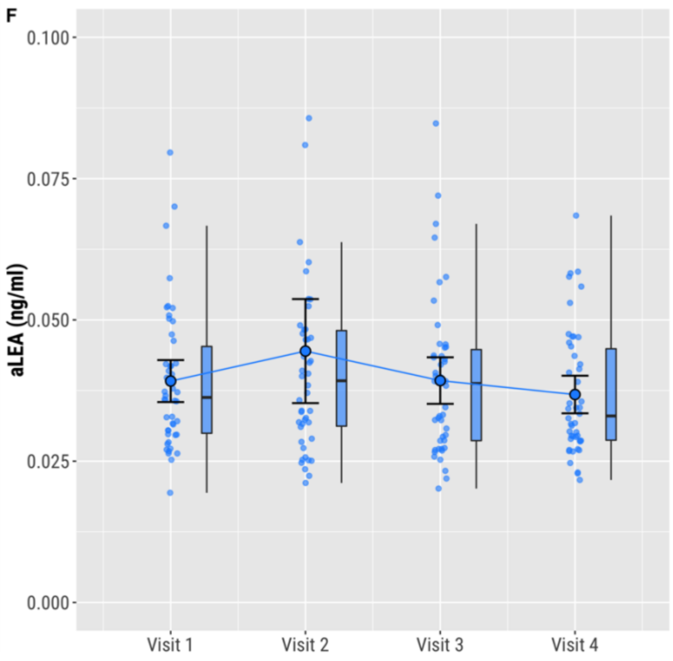

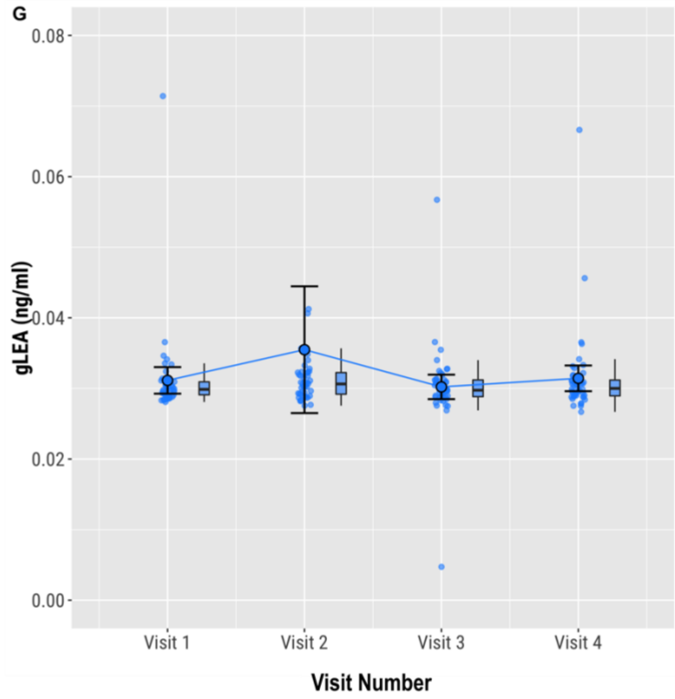

eFigure 5 continued.
Pre-inhalation plasma concentrations vs. visit number.

E. N-arachidonoyl-L-serine (ARA-S),
F. alpha-linolenoylethanolamide (aLEA),
G. gamma-linolenoylethanolamide (gLEA).

Circles show individual data points, diamonds show mean values and boxplots show median and interquartile range.

eTable 1. Pharmacokinetics for each contrast between CBD:THC ratios – Models 1 and 2

Statistically significant rows are presented in bold.

Contrast	Estimated marginal mean difference	Lower 95% CI	Upper 95% CI	p-value
	**THC AUC**			
0:1 - 1:1	-15.336	-32.679	2.008	0.287
0:1 - 2:1	-6.549	-23.893	10.794	0.872
0:1 - 3:1	-7.412	-24.756	9.931	0.825
1:1 - 2:1	8.786	-8.557	26.130	0.738
1:1 - 3:1	7.923	-9.420	25.267	0.794
2:1 - 3:1	-0.863	-18.207	16.480	1.000
	**THC AUC (adjusted for visit number)**			
0:1 - 1:1	-16.038	-33.514	1.437	0.256
0:1 - 2:1	-7.112	-24.571	10.347	0.845
0:1 - 3:1	-6.943	-24.495	10.609	0.856
1:1 - 2:1	8.927	-8.524	26.377	0.732
1:1 - 3:1	9.095	-8.499	26.689	0.726
2:1 - 3:1	0.169	-17.475	17.813	1.000
	**THC Peak**			
0:1 - 1:1	-9.255	-21.379	2.869	0.418
0:1 - 2:1	-0.085	-12.209	12.039	1.000
0:1 - 3:1	-1.013	-13.137	11.111	0.998
1:1 - 2:1	9.170	-2.954	21.294	0.426
1:1 - 3:1	8.242	-3.882	20.366	0.521
2:1 - 3:1	-0.928	-13.052	11.196	0.999
	**THC Peak (adjusted for visit number)**			
0:1 - 1:1	-9.255	-21.843	2.631	0.418
0:1 - 2:1	-0.085	-12.508	11.944	1.000
0:1 - 3:1	-1.013	-13.449	11.132	0.998
1:1 - 2:1	9.170	-2.896	21.544	0.426
1:1 - 3:1	8.242	-3.872	20.768	0.521
2:1 - 3:1	-0.876	-13.232	11.479	0.999

eTable 1 continued.

Contrast	Estimated marginal mean difference	Lower 95% CI	Upper 95% CI	p-value
	**CBD AUC**			
0:1 - 1:1	-123.709	-168.682	-78.736	9.088×10^{-7}
0:1 - 2:1	-244.322	-289.295	-199.349	1.565×10^{-14}
0:1 - 3:1	-345.036	-390.009	-300.063	$<2.22 \times 10^{-16}$
1:1 - 2:1	-120.613	-165.586	-75.640	1.722×10^{-6}
1:1 - 3:1	-221.327	-266.300	-176.354	2.975×10^{-14}
2:1 - 3:1	-100.714	-145.687	-55.741	8.148×10^{-5}
	**CBD AUC (adjusted for visit number)**			
0:1 - 1:1	-124.426	-169.361	-79.492	7.738×10^{-7}
0:1 - 2:1	-245.398	-290.354	-200.442	8.549×10^{-15}
0:1 - 3:1	-342.526	-387.656	-297.397	$<2.22 \times 10^{-16}$
1:1 - 2:1	-120.972	-165.893	-76.050	1.570×10^{-6}
1:1 - 3:1	-218.100	-263.368	-172.833	2.431×10^{-14}
2:1 - 3:1	-97.129	-142.478	-51.779	1.798×10^{-4}
	**CBD Peak**			
0:1 - 1:1	-61.543	-87.712	-35.374	3.197×10^{-5}
0:1 - 2:1	-109.768	-135.936	-83.599	2.828×10^{-13}
0:1 - 3:1	-153.819	-179.988	-127.650	1.110×10^{-16}
1:1 - 2:1	-48.225	-74.394	-22.056	0.002
1:1 - 3:1	-92.277	-118.445	-66.108	3.820×10^{-10}
2:1 - 3:1	-44.052	-70.221	-17.883	0.005
	**CBD Peak (adjusted for visit number)**			
0:1 - 1:1	-61.743	-87.989	-35.496	3.200×10^{-5}
0:1 - 2:1	-110.067	-136.327	-83.808	2.964×10^{-13}
0:1 - 3:1	-153.120	-179.480	-126.759	$<2.22 \times 10^{-16}$
1:1 - 2:1	-48.325	-74.564	-22.086	0.002
1:1 - 3:1	-91.377	-117.818	-64.936	8.214×10^{-10}
2:1 - 3:1	-43.052	-69.541	-16.563	0.007

eTable 1 continued.

Contrast	Estimated marginal mean difference	Lower 95% CI	Upper 95% CI	p-value
	**Anandamide AUC**			
0:1 - 1:1	0.007	-0.136	0.150	1.000
0:1 - 2:1	-0.062	-0.206	0.081	0.825
0:1 - 3:1	-0.015	-0.158	0.128	0.997
1:1 - 2:1	-0.069	-0.213	0.074	0.774
1:1 - 3:1	-0.022	-0.165	0.121	0.991
2:1 - 3:1	0.048	-0.096	0.191	0.913
	**Anandamide AUC (adjusted for baseline concentration, visit number and time between visits)**			
0:1 - 1:1	0.018	-0.098	0.134	0.990
0:1 - 2:1	-0.066	-0.182	0.050	0.673
0:1 - 3:1	0.007	-0.110	0.125	0.999
1:1 - 2:1	-0.084	-0.200	0.033	0.487
1:1 - 3:1	-0.011	-0.128	0.107	0.998
2:1 - 3:1	0.073	-0.046	0.192	0.617
	**Anandamide Peak**			
0:1 - 1:1	0.014	-0.028	0.057	0.912
0:1 - 2:1	-0.009	-0.052	0.033	0.971
0:1 - 3:1	-0.012	-0.055	0.030	0.942
1:1 - 2:1	-0.024	-0.066	0.019	0.690
1:1 - 3:1	-0.026	-0.069	0.016	0.612
2:1 - 3:1	-0.003	-0.045	0.040	0.999
	**Anandamide Peak (adjusted for baseline concentration, visit number and time between visits)**			
0:1 - 1:1	0.013	-0.029	0.055	0.927
0:1 - 2:1	-0.010	-0.052	0.031	0.962
0:1 - 3:1	-0.005	-0.047	0.037	0.995
1:1 - 2:1	-0.023	-0.065	0.019	0.692
1:1 - 3:1	-0.018	-0.060	0.024	0.828
2:1 - 3:1	0.005	-0.038	0.048	0.996

eTable 1 continued.

Contrast	Estimated marginal mean difference	Lower 95% CI	Upper 95% CI	p-value
	**2-AG AUC**			
0:1 - 1:1	-3.214	-11.042	4.613	0.849
0:1 - 2:1	-4.005	-11.833	3.822	0.743
0:1 - 3:1	-1.337	-9.164	6.491	0.987
1:1 - 2:1	-0.791	-8.619	7.037	0.997
1:1 - 3:1	1.878	-5.950	9.705	0.965
2:1 - 3:1	2.669	-5.159	10.496	0.907
	**2-AG AUC (adjusted for baseline concentration, visit number and time between visits)**			
0:1 - 1:1	1.068	-2.526	4.662	0.936
0:1 - 2:1	0.189	-3.391	3.770	1.000
0:1 - 3:1	3.068	-0.567	6.702	0.344
1:1 - 2:1	-0.879	-4.467	2.709	0.962
1:1 - 3:1	1.999	-1.611	5.610	0.693
2:1 - 3:1	2.879	-0.784	6.541	0.408
	**2-AG Peak**			
0:1 - 1:1	-1.656	-4.555	1.243	0.672
0:1 - 2:1	1.120	-1.778	4.018	0.870
0:1 - 3:1	0.463	-2.435	3.361	0.989
1:1 - 2:1	2.776	-0.108	5.661	0.231
1:1 - 3:1	2.119	-0.765	5.004	0.469
2:1 - 3:1	-0.657	-3.541	2.227	0.969
	**2-AG Peak (adjusted for baseline concentration, visit number and time between visits)**			
0:1 - 1:1	-1.711	-4.620	1.199	0.651
0:1 - 2:1	1.141	-1.758	4.041	0.864
0:1 - 3:1	0.180	-2.762	3.123	0.999
1:1 - 2:1	2.852	-0.053	5.757	0.216
1:1 - 3:1	1.891	-1.033	4.815	0.578
2:1 - 3:1	-0.961	-3.923	2.000	0.918

eTable 1 continued.

Contrast	Estimated marginal mean difference	Lower 95% CI	Upper 95% CI	p-value
	**Arachidonic acid AUC**			
0:1 - 1:1	36.986	-148.669	222.642	0.979
0:1 - 2:1	-42.727	-228.383	142.929	0.969
0:1 - 3:1	83.001	-102.655	268.657	0.813
1:1 - 2:1	-79.713	-265.369	105.942	0.831
1:1 - 3:1	46.015	-139.641	231.670	0.961
2:1 - 3:1	125.728	-59.928	311.384	0.540
	**Arachidonic acid AUC (adjusted for baseline concentration, visit number and time between visits)**			
0:1 - 1:1	17.069	-132.965	167.104	0.996
0:1 - 2:1	-68.190	-217.688	81.309	0.804
0:1 - 3:1	29.306	-123.244	181.856	0.981
1:1 - 2:1	-85.259	-235.767	65.250	0.677
1:1 - 3:1	12.237	-139.517	163.990	0.999
2:1 - 3:1	97.495	-56.136	251.127	0.593
	**Arachidonic acid Peak**			
0:1 - 1:1	9.139	-48.117	66.395	0.989
0:1 - 2:1	-21.807	-79.118	35.503	0.875
0:1 - 3:1	36.819	-20.516	94.154	0.584
1:1 - 2:1	-30.946	-88.219	26.327	0.709
1:1 - 3:1	27.680	-29.609	84.970	0.775
2:1 - 3:1	58.626	1.377	115.876	0.184
	**Arachidonic acid Peak (adjusted for baseline concentration, visit number and time between visits)**			
0:1 - 1:1	8.911	-48.980	66.802	0.990
0:1 - 2:1	-19.579	-77.275	38.118	0.908
0:1 - 3:1	33.525	-25.289	92.339	0.673
1:1 - 2:1	-28.489	-86.536	29.557	0.766
1:1 - 3:1	24.614	-33.931	83.160	0.839
2:1 - 3:1	53.103	-6.114	112.321	0.291

eTable 1 continued.

Contrast	Estimated marginal mean difference	Lower 95% CI	Upper 95% CI	p-value
	**DEA AUC**			
0:1 - 1:1	0.013	-0.020	0.045	0.864
0:1 - 2:1	0.014	-0.018	0.047	0.814
0:1 - 3:1	0.005	-0.027	0.037	0.990
1:1 - 2:1	0.002	-0.031	0.034	1.000
1:1 - 3:1	-0.008	-0.040	0.025	0.965
2:1 - 3:1	-0.009	-0.042	0.023	0.938
	**DEA AUC (adjusted for baseline concentration, visit number and time between visits)**			
0:1 - 1:1	0.013	-0.006	0.031	0.535
0:1 - 2:1	0.009	-0.010	0.027	0.785
0:1 - 3:1	0.015	-0.004	0.034	0.393
1:1 - 2:1	-0.004	-0.023	0.015	0.976
1:1 - 3:1	0.002	-0.016	0.021	0.994
2:1 - 3:1	0.006	-0.013	0.025	0.915
	**DEA Peak**			
0:1 - 1:1	0.003	-0.006	0.012	0.894
0:1 - 2:1	0.001	-0.008	0.010	0.998
0:1 - 3:1	0.000	-0.009	0.009	1.000
1:1 - 2:1	-0.002	-0.011	0.007	0.952
1:1 - 3:1	-0.004	-0.013	0.006	0.867
2:1 - 3:1	-0.001	-0.010	0.008	0.995
	**DEA Peak (adjusted for baseline concentration, visit number and time between visits)**			
0:1 - 1:1	0.004	-0.005	0.013	0.848
0:1 - 2:1	0.001	-0.008	0.010	0.998
0:1 - 3:1	0.002	-0.008	0.011	0.987
1:1 - 2:1	-0.003	-0.012	0.006	0.926
1:1 - 3:1	-0.002	-0.011	0.007	0.966
2:1 - 3:1	0.001	-0.009	0.010	0.999

eTable 1 continued.

Contrast	Estimated marginal mean difference	Lower 95% CI	Upper 95% CI	p-value
	**OEA AUC**			
0:1 - 1:1	0.312	-0.469	1.093	0.859
0:1 - 2:1	0.048	-0.733	0.829	0.999
0:1 - 3:1	0.357	-0.424	1.138	0.802
1:1 - 2:1	-0.264	-1.045	0.517	0.908
1:1 - 3:1	0.045	-0.736	0.826	0.999
2:1 - 3:1	0.310	-0.471	1.091	0.861
	**OEA AUC (adjusted for baseline concentration, visit number and time between visits)**			
0:1 - 1:1	0.412	-0.242	1.066	0.599
0:1 - 2:1	-0.008	-0.659	0.643	1.000
0:1 - 3:1	0.264	-0.398	0.927	0.859
1:1 - 2:1	-0.420	-1.078	0.237	0.587
1:1 - 3:1	-0.148	-0.809	0.514	0.971
2:1 - 3:1	0.273	-0.396	0.941	0.851
	**OEA Peak**			
0:1 - 1:1	0.188	-0.028	0.405	0.315
0:1 - 2:1	0.015	-0.201	0.231	0.999
0:1 - 3:1	0.047	-0.169	0.263	0.974
1:1 - 2:1	-0.174	-0.390	0.043	0.390
1:1 - 3:1	-0.142	-0.358	0.075	0.568
2:1 - 3:1	0.032	-0.184	0.248	0.991
	**OEA Peak (adjusted for baseline concentration, visit number and time between visits)**			
0:1 - 1:1	0.192	-0.028	0.412	0.312
0:1 - 2:1	0.019	-0.200	0.237	0.998
0:1 - 3:1	0.059	-0.163	0.281	0.954
1:1 - 2:1	-0.173	-0.394	0.048	0.410
1:1 - 3:1	-0.133	-0.355	0.089	0.635
2:1 - 3:1	0.040	-0.184	0.264	0.985

eTable 1 continued.

Contrast	Estimated marginal mean difference	Lower 95% CI	Upper 95% CI	p-value
	**SEA AUC**			
0:1 - 1:1	0.431	-0.583	1.445	0.835
0:1 - 2:1	0.198	-0.815	1.212	0.980
0:1 - 3:1	0.472	-0.541	1.486	0.793
1:1 - 2:1	-0.233	-1.246	0.781	0.969
1:1 - 3:1	0.041	-0.972	1.055	1.000
2:1 - 3:1	0.274	-0.740	1.288	0.951
	**SEA AUC (adjusted for baseline concentration, visit number and time between visits)**			
0:1 - 1:1	0.164	-0.252	0.581	0.863
0:1 - 2:1	0.056	-0.358	0.471	0.993
0:1 - 3:1	0.295	-0.125	0.716	0.509
1:1 - 2:1	-0.108	-0.525	0.309	0.956
1:1 - 3:1	0.131	-0.289	0.551	0.927
2:1 - 3:1	0.239	-0.186	0.664	0.682
	**SEA Peak**			
0:1 - 1:1	0.139	-0.114	0.392	0.700
0:1 - 2:1	0.003	-0.250	0.256	1.000
0:1 - 3:1	0.109	-0.144	0.363	0.828
1:1 - 2:1	-0.136	-0.389	0.118	0.715
1:1 - 3:1	-0.029	-0.282	0.224	0.996
2:1 - 3:1	0.106	-0.147	0.359	0.840
	**SEA Peak (adjusted for baseline concentration, visit number and time between visits)**			
0:1 - 1:1	0.142	-0.115	0.398	0.694
0:1 - 2:1	0.013	-0.242	0.268	1.000
0:1 - 3:1	0.103	-0.156	0.362	0.861
1:1 - 2:1	-0.129	-0.386	0.128	0.754
1:1 - 3:1	-0.039	-0.298	0.220	0.991
2:1 - 3:1	0.090	-0.172	0.351	0.905

eTable 1 continued.

Contrast	Estimated marginal mean difference	Lower 95% CI	Upper 95% CI	p-value
	**ARA-S AUC**			
0:1 - 1:1	-0.008	-0.053	0.036	0.983
0:1 - 2:1	-0.002	-0.046	0.043	1.000
0:1 - 3:1	0.004	-0.041	0.048	0.998
1:1 - 2:1	0.007	-0.038	0.051	0.991
1:1 - 3:1	0.012	-0.032	0.056	0.951
2:1 - 3:1	0.005	-0.039	0.050	0.995
	**ARA-S AUC (adjusted for baseline concentration, visit number and time between visits)**			
0:1 - 1:1	0.023	-0.004	0.050	0.352
0:1 - 2:1	0.011	-0.016	0.038	0.838
0:1 - 3:1	0.014	-0.013	0.042	0.723
1:1 - 2:1	-0.011	-0.039	0.016	0.839
1:1 - 3:1	-0.008	-0.036	0.019	0.932
2:1 - 3:1	0.003	-0.025	0.031	0.996
	**ARA-S Peak**			
0:1 - 1:1	0.007	-0.007	0.021	0.758
0:1 - 2:1	-0.002	-0.016	0.012	0.991
0:1 - 3:1	0.002	-0.012	0.016	0.987
1:1 - 2:1	-0.009	-0.023	0.005	0.575
1:1 - 3:1	-0.005	-0.019	0.009	0.913
2:1 - 3:1	0.004	-0.010	0.018	0.923
	**ARA-S Peak (adjusted for baseline concentration, visit number and time between visits)**			
0:1 - 1:1	0.007	-0.007	0.021	0.764
0:1 - 2:1	-0.002	-0.017	0.012	0.986
0:1 - 3:1	0.004	-0.011	0.018	0.957
1:1 - 2:1	-0.009	-0.024	0.005	0.554
1:1 - 3:1	-0.003	-0.018	0.011	0.968
2:1 - 3:1	0.006	-0.008	0.021	0.836

eTable 1 continued.

Contrast	Estimated marginal mean difference	Lower 95% CI	Upper 95% CI	p-value
	**aLEA AUC**			
0:1 - 1:1	-0.009	-0.038	0.021	0.939
0:1 - 2:1	-0.022	-0.051	0.007	0.457
0:1 - 3:1	-0.006	-0.036	0.023	0.972
1:1 - 2:1	-0.013	-0.043	0.016	0.808
1:1 - 3:1	0.002	-0.027	0.031	0.999
2:1 - 3:1	0.015	-0.014	0.045	0.728
	**aLEA AUC (adjusted for baseline concentration, visit number and time between visits)**			
0:1 - 1:1	0.001	-0.017	0.019	1.000
0:1 - 2:1	-0.012	-0.029	0.006	0.554
0:1 - 3:1	-0.003	-0.021	0.015	0.985
1:1 - 2:1	-0.013	-0.030	0.005	0.492
1:1 - 3:1	-0.004	-0.022	0.014	0.968
2:1 - 3:1	0.009	-0.010	0.027	0.787
	**aLEA Peak**			
0:1 - 1:1	0.001	-0.005	0.007	0.990
0:1 - 2:1	-0.004	-0.009	0.002	0.626
0:1 - 3:1	-0.003	-0.009	0.003	0.726
1:1 - 2:1	-0.004	-0.010	0.001	0.432
1:1 - 3:1	-0.004	-0.010	0.002	0.535
2:1 - 3:1	0.000	-0.005	0.006	0.998
	**aLEA Peak (adjusted for baseline concentration, visit number and time between visits)**			
0:1 - 1:1	0.001	-0.005	0.007	0.982
0:1 - 2:1	-0.003	-0.009	0.002	0.652
0:1 - 3:1	-0.003	-0.009	0.003	0.765
1:1 - 2:1	-0.005	-0.011	0.001	0.424
1:1 - 3:1	-0.004	-0.010	0.002	0.537
2:1 - 3:1	0.001	-0.006	0.007	0.998

eTable 1 continued.

Contrast	Estimated marginal mean difference	Lower 95% CI	Upper 95% CI	p-value
	**gLEA AUC**			
0:1 - 1:1	-0.011	-0.037	0.014	0.810
0:1 - 2:1	-0.010	-0.035	0.015	0.866
0:1 - 3:1	-0.009	-0.034	0.016	0.892
1:1 - 2:1	0.001	-0.024	0.027	0.999
1:1 - 3:1	0.002	-0.023	0.028	0.998
2:1 - 3:1	0.001	-0.024	0.026	1.000
	**gLEA AUC (adjusted for baseline concentration, visit number and time between visits)**			
0:1 - 1:1	0.002	-0.006	0.009	0.963
0:1 - 2:1	0.001	-0.006	0.009	0.979
0:1 - 3:1	0.002	-0.006	0.009	0.974
1:1 - 2:1	0.000	-0.008	0.007	1.000
1:1 - 3:1	0.000	-0.008	0.007	1.000
2:1 - 3:1	0.000	-0.008	0.008	1.000
	**gLEA Peak**			
0:1 - 1:1	0.000	-0.002	0.002	1.000
0:1 - 2:1	0.001	-0.001	0.003	0.886
0:1 - 3:1	0.002	-0.001	0.004	0.452
1:1 - 2:1	0.001	-0.001	0.003	0.907
1:1 - 3:1	0.001	-0.001	0.004	0.483
2:1 - 3:1	0.001	-0.001	0.003	0.874
	**gLEA Peak (adjusted for baseline concentration, visit number and time between visits)**			
0:1 - 1:1	0.000	-0.002	0.002	0.998
0:1 - 2:1	0.001	-0.001	0.003	0.900
0:1 - 3:1	0.002	0.000	0.004	0.246
1:1 - 2:1	0.001	-0.002	0.003	0.957
1:1 - 3:1	0.002	0.000	0.004	0.327
2:1 - 3:1	0.001	-0.001	0.003	0.640

eTable 2. Plasma concentrations vs. time following 10mg THC, 0mg CBD (0:1 ratio) – Model 3a

Statistically significant rows are presented in bold.
Pre = pre-inhalation plasma sample.

Contrast	Estimated marginal mean difference	Lower 95% CI	Upper 95% CI	p-value
	**Anandamide (ng/ml)**			
Pre – 0min	-0.040	-0.070	-0.010	0.069
Pre – 5min	0.003	-0.027	0.033	1.000
Pre – 15min	0.011	-0.019	0.041	0.949
Pre – 90min	0.019	-0.011	0.049	0.735
**0min – 5min**	**0.043**	**0.013**	**0.073**	**0.040**
**0min – 15min**	**0.051**	**0.021**	**0.081**	**0.008**
**0min – 90min**	**0.059**	**0.029**	**0.089**	**0.001**
5min – 15min	0.008	-0.022	0.038	0.985
5min – 90min	0.016	-0.014	0.046	0.845
15min – 90min	0.008	-0.022	0.038	0.988
	**Anandamide (ng/ml) (adjusted for visit number and time between visits)**			
Pre – 0min	-0.040	-0.070	-0.010	0.069
Pre – 5min	0.003	-0.027	0.033	1.000
Pre – 15min	0.011	-0.019	0.041	0.949
Pre – 90min	0.019	-0.011	0.049	0.735
**0min – 5min**	**0.043**	**0.013**	**0.073**	**0.040**
**0min – 15min**	**0.051**	**0.021**	**0.081**	**0.008**
**0min – 90min**	**0.059**	**0.029**	**0.089**	**0.001**
5min – 15min	0.008	-0.022	0.038	0.985
5min – 90min	0.016	-0.014	0.046	0.845
15min – 90min	0.008	-0.022	0.038	0.988

eTable 2 continued.

Contrast	Estimated marginal mean difference	Lower 95% CI	Upper 95% CI	p-value
	**2-AG (ng/ml)**			
Pre – 0min	0.513	-1.451	2.478	0.986
Pre – 5min	0.621	-1.343	2.586	0.971
Pre – 15min	0.891	-1.073	2.855	0.898
Pre – 90min	0.291	-1.673	2.255	0.998
0min – 5min	0.108	-1.856	2.072	1.000
0min – 15min	0.378	-1.587	2.342	0.996
0min – 90min	-0.222	-2.187	1.742	0.999
5min – 15min	0.270	-1.695	2.234	0.999
5min – 90min	-0.330	-2.295	1.634	0.997
15min – 90min	-0.600	-2.564	1.364	0.975
	**2-AG (ng/ml) (adjusted for visit number and time between visits)**			
Pre – 0min	0.513	-1.451	2.478	0.986
Pre – 5min	0.621	-1.343	2.586	0.971
Pre – 15min	0.891	-1.073	2.855	0.898
Pre – 90min	0.291	-1.673	2.255	0.998
0min – 5min	0.108	-1.856	2.072	1.000
0min – 15min	0.378	-1.587	2.342	0.996
0min – 90min	-0.222	-2.187	1.742	0.999
5min – 15min	0.270	-1.695	2.234	0.999
5min – 90min	-0.330	-2.295	1.634	0.997
15min – 90min	-0.600	-2.564	1.364	0.975

eTable 2 continued.

Contrast	Estimated marginal mean difference	Lower 95% CI	Upper 95% CI	p-value
	**Arachidonic acid (ng/ml)**			
Pre – 0min	-33.180	-74.904	8.543	0.519
Pre – 5min	-46.043	-87.767	-4.320	0.193
Pre – 15min	-5.436	-47.159	36.287	0.999
Pre – 90min	3.859	-37.865	45.582	1.000
0min – 5min	-12.863	-54.586	28.860	0.974
0min – 15min	27.744	-13.979	69.468	0.684
0min – 90min	37.039	-4.684	78.762	0.405
5min – 15min	40.607	-1.116	82.331	0.310
5min – 90min	49.902	8.179	91.626	0.131
15min – 90min	9.295	-32.429	51.018	0.992
	**Arachidonic acid (ng/ml) (adjusted for visit number and time between visits)**			
Pre – 0min	-33.180	-74.904	8.543	0.519
Pre – 5min	-46.043	-87.767	-4.320	0.193
Pre – 15min	-5.436	-47.159	36.287	0.999
Pre – 90min	3.859	-37.865	45.582	1.000
0min – 5min	-12.863	-54.586	28.860	0.974
0min – 15min	27.744	-13.979	69.468	0.684
0min – 90min	37.039	-4.684	78.762	0.405
5min – 15min	40.607	-1.116	82.331	0.310
5min – 90min	49.902	8.179	91.626	0.131
15min – 90min	9.295	-32.429	51.018	0.992

eTable 2 continued.

Contrast	Estimated marginal mean difference	Lower 95% CI	Upper 95% CI	p-value
	**DEA (ng/ml)**			
**Pre – 0min**	**-0.013**	**-0.020**	**-0.005**	**0.011**
Pre – 5min	-0.001	-0.009	0.006	0.998
Pre – 15min	-0.003	-0.011	0.005	0.952
Pre – 90min	0.003	-0.004	0.011	0.907
**0min – 5min**	**0.012**	**0.004**	**0.019**	**0.029**
0min – 15min	0.010	0.002	0.018	0.084
**0min – 90min**	**0.016**	**0.008**	**0.024**	**0.000**
5min – 15min	-0.002	-0.009	0.006	0.995
5min – 90min	0.005	-0.003	0.012	0.756
15min – 90min	0.006	-0.001	0.014	0.504
	**DEA (ng/ml) (adjusted for visit number and time between visits)**			
**Pre – 0min**	**-0.013**	**-0.020**	**-0.005**	**0.011**
Pre – 5min	-0.001	-0.009	0.006	0.998
Pre – 15min	-0.003	-0.011	0.005	0.952
Pre – 90min	0.003	-0.004	0.011	0.907
**0min – 5min**	**0.012**	**0.004**	**0.019**	**0.029**
0min – 15min	0.010	0.002	0.018	0.084
**0min – 90min**	**0.016**	**0.008**	**0.024**	**0.000**
5min – 15min	-0.002	-0.009	0.006	0.995
5min – 90min	0.005	-0.003	0.012	0.756
15min – 90min	0.006	-0.001	0.014	0.504

eTable 2 continued.

Contrast	Estimated marginal mean difference	Lower 95% CI	Upper 95% CI	p-value
	**OEA (ng/ml)**			
Pre – 0min	-0.250	-0.432	-0.068	0.057
Pre – 5min	-0.109	-0.291	0.073	0.760
Pre – 15min	0.020	-0.162	0.202	0.999
Pre – 90min	0.066	-0.116	0.248	0.952
0min – 5min	0.141	-0.041	0.322	0.548
**0min – 15min**	**0.270**	**0.088**	**0.452**	**0.031**
**0min – 90min**	**0.316**	**0.134**	**0.498**	**0.007**
5min – 15min	0.129	-0.053	0.311	0.627
5min – 90min	0.175	-0.007	0.357	0.320
15min – 90min	0.046	-0.136	0.228	0.987
	**OEA (ng/ml) (adjusted for visit number and time between visits)**			
Pre – 0min	-0.250	-0.432	-0.068	0.057
Pre – 5min	-0.109	-0.291	0.073	0.760
Pre – 15min	0.020	-0.162	0.202	0.999
Pre – 90min	0.066	-0.116	0.248	0.952
0min – 5min	0.141	-0.041	0.322	0.548
**0min – 15min**	**0.270**	**0.088**	**0.452**	**0.031**
**0min – 90min**	**0.316**	**0.134**	**0.498**	**0.007**
5min – 15min	0.129	-0.053	0.311	0.627
5min – 90min	0.175	-0.007	0.357	0.320
15min – 90min	0.046	-0.136	0.228	0.987

eTable 2 continued.

Contrast	Estimated marginal mean difference	Lower 95% CI	Upper 95% CI	p-value
	**SEA (ng/ml)**			
Pre – 0min	-0.156	-0.387	0.075	0.673
Pre – 5min	-0.007	-0.238	0.224	1.000
Pre – 15min	-0.013	-0.244	0.218	1.000
Pre – 90min	0.030	-0.201	0.261	0.999
0min – 5min	0.149	-0.082	0.380	0.709
0min – 15min	0.143	-0.088	0.374	0.741
0min – 90min	0.186	-0.045	0.417	0.507
5min – 15min	-0.006	-0.237	0.225	1.000
5min – 90min	0.037	-0.194	0.268	0.998
15min – 90min	0.043	-0.188	0.274	0.996
	**SEA (ng/ml) (adjusted for visit number and time between visits)**			
Pre – 0min	-0.156	-0.387	0.075	0.673
Pre – 5min	-0.007	-0.238	0.224	1.000
Pre – 15min	-0.013	-0.244	0.218	1.000
Pre – 90min	0.030	-0.201	0.261	0.999
0min – 5min	0.149	-0.082	0.380	0.709
0min – 15min	0.143	-0.088	0.374	0.741
0min – 90min	0.186	-0.045	0.417	0.507
5min – 15min	-0.006	-0.237	0.225	1.000
5min – 90min	0.037	-0.194	0.268	0.998
15min – 90min	0.043	-0.188	0.274	0.996

eTable 2 continued.

Contrast	Estimated marginal mean difference	Lower 95% CI	Upper 95% CI	p-value
	**ARA-S (ng/ml)**			
Pre – 0min	-0.009	-0.021	0.003	0.552
Pre – 5min	-0.006	-0.018	0.006	0.883
Pre – 15min	-0.001	-0.013	0.011	1.000
Pre – 90min	-0.001	-0.013	0.012	1.000
0min – 5min	0.004	-0.009	0.016	0.978
0min – 15min	0.008	-0.004	0.020	0.657
0min – 90min	0.009	-0.003	0.021	0.614
5min – 15min	0.005	-0.007	0.017	0.938
5min – 90min	0.005	-0.007	0.017	0.918
15min – 90min	0.000	-0.012	0.013	1.000
	**ARA-S (ng/ml) (adjusted for visit number and time between visits)**			
Pre – 0min	-0.009	-0.021	0.003	0.552
Pre – 5min	-0.006	-0.018	0.006	0.883
Pre – 15min	-0.001	-0.013	0.011	1.000
Pre – 90min	-0.001	-0.013	0.012	1.000
0min – 5min	0.004	-0.009	0.016	0.978
0min – 15min	0.008	-0.004	0.020	0.657
0min – 90min	0.009	-0.003	0.021	0.614
5min – 15min	0.005	-0.007	0.017	0.938
5min – 90min	0.005	-0.007	0.017	0.918
15min – 90min	0.000	-0.012	0.013	1.000

eTable 2 continued.

Contrast	Estimated marginal mean difference	Lower 95% CI	Upper 95% CI	p-value
	**aLEA (ng/ml)**			
Pre – 0min	0.004	-0.003	0.011	0.820
Pre – 5min	0.004	-0.003	0.010	0.857
Pre – 15min	0.004	-0.003	0.011	0.817
Pre – 90min	0.007	0.000	0.014	0.265
0min – 5min	0.000	-0.007	0.007	1.000
0min – 15min	0.000	-0.007	0.007	1.000
0min – 90min	0.003	-0.004	0.010	0.882
5min – 15min	0.000	-0.007	0.007	1.000
5min – 90min	0.004	-0.003	0.011	0.849
15min – 90min	0.003	-0.004	0.010	0.884
	**aLEA (ng/ml) (adjusted for visit number and time between visits)**			
Pre – 0min	0.004	-0.003	0.011	0.820
Pre – 5min	0.004	-0.003	0.010	0.857
Pre – 15min	0.004	-0.003	0.011	0.817
Pre – 90min	0.007	0.000	0.014	0.265
0min – 5min	0.000	-0.007	0.007	1.000
0min – 15min	0.000	-0.007	0.007	1.000
0min – 90min	0.003	-0.004	0.010	0.882
5min – 15min	0.000	-0.007	0.007	1.000
5min – 90min	0.004	-0.003	0.011	0.849
15min – 90min	0.003	-0.004	0.010	0.884

eTable 2 continued.

Contrast	Estimated marginal mean difference	Lower 95% CI	Upper 95% CI	p-value
	**gLEA (ng/ml)**			
Pre – 0min	0.005	-0.002	0.011	0.625
Pre – 5min	0.004	-0.003	0.010	0.817
Pre – 15min	0.001	-0.005	0.008	0.995
Pre – 90min	0.005	-0.001	0.012	0.516
0min – 5min	-0.001	-0.008	0.005	0.998
0min – 15min	-0.003	-0.010	0.003	0.849
0min – 90min	0.001	-0.006	0.007	1.000
5min – 15min	-0.002	-0.009	0.004	0.959
5min – 90min	0.002	-0.005	0.008	0.988
15min – 90min	0.004	-0.003	0.010	0.762
	**gLEA (ng/ml) (adjusted for visit number and time between visits)**			
Pre – 0min	0.005	-0.002	0.011	0.627
Pre – 5min	0.004	-0.003	0.010	0.818
Pre – 15min	0.001	-0.005	0.008	0.995
Pre – 90min	0.005	-0.001	0.012	0.518
0min – 5min	-0.001	-0.008	0.005	0.998
0min – 15min	-0.003	-0.010	0.003	0.850
0min – 90min	0.001	-0.006	0.007	1.000
5min – 15min	-0.002	-0.009	0.004	0.959
5min – 90min	0.002	-0.005	0.008	0.988
15min – 90min	0.004	-0.003	0.011	0.764

eTable 3. Plasma concentrations vs. time following 10mg THC (all CBD:THC ratios) – Model 3b

Statistically significant rows are presented in bold.
Pre = pre-inhalation plasma sample.

Contrast	Estimated marginal mean difference	Lower 95% CI	Upper 95% CI	p-value
	**Anandamide (ng/ml)**			
**Pre – 0min**	**-0.044**	**-0.064**	**-0.025**	**0.000**
Pre – 5min	0.003	-0.017	0.022	0.999
Pre – 15min	0.001	-0.019	0.020	1.000
Pre – 90min	0.017	-0.002	0.037	0.420
**0min – 5min**	**0.047**	**0.028**	**0.067**	**0.000**
**0min – 15min**	**0.045**	**0.025**	**0.065**	**0.000**
**0min – 90min**	**0.061**	**0.042**	**0.081**	**0.000**
5min – 15min	-0.002	-0.022	0.017	1.000
5min – 90min	0.014	-0.005	0.034	0.604
15min – 90min	0.016	-0.003	0.036	0.465
	**Anandamide (ng/ml) (adjusted for CBD:THC ratio, visit number and time between visits)**			
**Pre – 0min**	**-0.042**	**-0.062**	**-0.023**	**0.000**
Pre – 5min	0.003	-0.016	0.022	0.998
Pre – 15min	0.002	-0.018	0.021	1.000
Pre – 90min	0.018	-0.001	0.037	0.349
**0min – 5min**	**0.045**	**0.026**	**0.065**	**0.000**
**0min – 15min**	**0.044**	**0.025**	**0.063**	**0.000**
**0min – 90min**	**0.060**	**0.041**	**0.080**	**0.000**
5min – 15min	-0.001	-0.021	0.018	1.000
5min – 90min	0.015	-0.004	0.034	0.547
15min – 90min	0.016	-0.003	0.036	0.457

eTable 3 continued.

Contrast	Estimated marginal mean difference	Lower 95% CI	Upper 95% CI	p-value
	**2-AG (ng/ml)**			
Pre – 0min	0.030	-0.986	1.046	1.000
Pre – 5min	0.251	-0.765	1.267	0.989
Pre – 15min	0.083	-0.933	1.099	1.000
Pre – 90min	-0.521	-1.537	0.496	0.853
0min – 5min	0.221	-0.796	1.237	0.993
0min – 15min	0.053	-0.963	1.069	1.000
0min – 90min	-0.551	-1.567	0.465	0.825
5min – 15min	-0.168	-1.184	0.848	0.998
5min – 90min	-0.771	-1.787	0.245	0.569
15min – 90min	-0.603	-1.619	0.413	0.771
	**2-AG (ng/ml) (adjusted for CBD:THC ratio, visit number and time between visits)**			
Pre – 0min	0.030	-0.985	1.045	1.000
Pre – 5min	0.251	-0.764	1.265	0.989
Pre – 15min	0.083	-0.932	1.098	1.000
Pre – 90min	-0.521	-1.535	0.494	0.852
0min – 5min	0.221	-0.794	1.235	0.993
0min – 15min	0.053	-0.962	1.067	1.000
0min – 90min	-0.551	-1.565	0.464	0.824
5min – 15min	-0.168	-1.183	0.847	0.998
5min – 90min	-0.771	-1.786	0.244	0.568
15min – 90min	-0.603	-1.618	0.411	0.770

eTable 3 continued.

Contrast	Estimated marginal mean difference	Lower 95% CI	Upper 95% CI	p-value
	**Arachidonic acid (ng/ml)**			
Pre – 0min	-28.446	-56.267	-0.625	0.263
Pre – 5min	-34.638	-62.458	-6.817	0.105
Pre – 15min	-6.587	-34.408	21.233	0.990
Pre – 90min	15.642	-12.179	43.463	0.805
0min – 5min	-6.192	-34.012	21.629	0.992
0min – 15min	21.859	-5.962	49.679	0.535
**0min – 90min**	**44.088**	**16.267**	**71.909**	**0.016**
5min – 15min	28.050	0.229	55.871	0.277
**5min – 90min**	**50.279**	**22.459**	**78.100**	**0.004**
15min – 90min	22.229	-5.592	50.050	0.518
	**Arachidonic acid (ng/ml) (adjusted for CBD:THC ratio, visit number and time between visits)**			
Pre – 0min	-27.334	-54.956	0.287	0.296
Pre – 5min	-34.263	-61.885	-6.641	0.107
Pre – 15min	-5.838	-33.460	21.784	0.994
Pre – 90min	16.478	-11.144	44.100	0.768
0min – 5min	-6.929	-34.550	20.693	0.988
0min – 15min	21.496	-6.126	49.118	0.545
**0min – 90min**	**43.812**	**16.191**	**71.434**	**0.016**
5min – 15min	28.425	0.803	56.047	0.257
**5min – 90min**	**50.741**	**23.119**	**78.363**	**0.003**
15min – 90min	22.316	-5.306	49.938	0.507

eTable 3 continued.

Contrast	Estimated marginal mean difference	Lower 95% CI	Upper 95% CI	p-value
	**DEA (ng/ml)**			
**Pre – 0min**	**-0.012**	**-0.016**	**-0.008**	**0.000**
Pre – 5min	0.002	-0.002	0.006	0.805
Pre – 15min	0.001	-0.003	0.005	0.995
Pre – 90min	0.003	-0.001	0.007	0.497
**0min – 5min**	**0.014**	**0.010**	**0.018**	**0.000**
**0min – 15min**	**0.013**	**0.009**	**0.017**	**0.000**
**0min – 90min**	**0.015**	**0.011**	**0.019**	**0.000**
5min – 15min	-0.001	-0.005	0.003	0.954
5min – 90min	0.001	-0.003	0.005	0.988
15min – 90min	0.002	-0.002	0.006	0.748
	**DEA (ng/ml) (adjusted for CBD:THC ratio, visit number and time between visits)**			
**Pre – 0min**	**-0.012**	**-0.016**	**-0.008**	**0.000**
Pre – 5min	0.002	-0.002	0.006	0.795
Pre – 15min	0.001	-0.003	0.005	0.995
Pre – 90min	0.003	0.000	0.007	0.412
**0min – 5min**	**0.014**	**0.010**	**0.018**	**0.000**
**0min – 15min**	**0.012**	**0.008**	**0.016**	**0.000**
**0min – 90min**	**0.015**	**0.011**	**0.019**	**0.000**
5min – 15min	-0.001	-0.005	0.002	0.952
5min – 90min	0.001	-0.003	0.005	0.973
15min – 90min	0.003	-0.001	0.007	0.669

eTable 3 continued.

Contrast	Estimated marginal mean difference	Lower 95% CI	Upper 95% CI	p-value
	**OEA (ng/ml)**			
**Pre – 0min**	**-0.186**	**-0.296**	**-0.077**	**0.008**
Pre – 5min	-0.023	-0.133	0.086	0.994
Pre – 15min	0.033	-0.076	0.142	0.976
Pre – 90min	0.096	-0.013	0.206	0.418
**0min – 5min**	**0.163**	**0.054**	**0.272**	**0.029**
**0min – 15min**	**0.219**	**0.110**	**0.329**	**0.001**
**0min – 90min**	**0.282**	**0.173**	**0.392**	**0.000**
5min – 15min	0.056	-0.053	0.166	0.850
5min – 90min	0.119	0.010	0.229	0.203
15min – 90min	0.063	-0.046	0.172	0.790
	**OEA (ng/ml) (adjusted for CBD:THC ratio, visit number and time between visits)**			
**Pre – 0min**	**-0.184**	**-0.293**	**-0.076**	**0.008**
Pre – 5min	-0.024	-0.133	0.084	0.992
Pre – 15min	0.030	-0.079	0.138	0.984
Pre – 90min	0.095	-0.013	0.204	0.420
**0min – 5min**	**0.160**	**0.051**	**0.269**	**0.032**
**0min – 15min**	**0.214**	**0.105**	**0.323**	**0.001**
**0min – 90min**	**0.280**	**0.171**	**0.388**	**0.000**
5min – 15min	0.054	-0.055	0.163	0.866
5min – 90min	0.120	0.011	0.228	0.194
15min – 90min	0.066	-0.043	0.174	0.757

eTable 3 continued.

Contrast	Estimated marginal mean difference	Lower 95% CI	Upper 95% CI	p-value
	**SEA (ng/ml)**			
Pre – 0min	-0.056	-0.163	0.052	0.847
Pre – 5min	0.065	-0.043	0.172	0.762
Pre – 15min	0.058	-0.049	0.166	0.822
Pre – 90min	0.064	-0.043	0.171	0.770
0min – 5min	0.120	0.013	0.227	0.181
0min – 15min	0.114	0.007	0.221	0.226
0min – 90min	0.119	0.012	0.227	0.186
5min – 15min	-0.006	-0.113	0.101	1.000
5min – 90min	-0.001	-0.108	0.106	1.000
15min – 90min	0.005	-0.102	0.113	1.000
	**SEA (ng/ml) (adjusted for CBD:THC ratio, visit number and time between visits)**			
Pre – 0min	-0.056	-0.168	0.046	0.846
Pre – 5min	0.065	-0.052	0.162	0.761
Pre – 15min	0.058	-0.059	0.155	0.821
Pre – 90min	0.064	-0.053	0.161	0.769
0min – 5min	0.120	0.010	0.224	0.179
0min – 15min	0.114	0.003	0.217	0.224
0min – 90min	0.119	0.008	0.222	0.185
5min – 15min	-0.006	-0.114	0.100	1.000
5min – 90min	-0.001	-0.109	0.105	1.000
15min – 90min	0.005	-0.102	0.112	1.000

eTable 3 continued.

Contrast	Estimated marginal mean difference	Lower 95% CI	Upper 95% CI	p-value
	**ARA-S (ng/ml) (adjusted for CBD:THC ratio and visit number)**			
**Pre – 0min**	**-0.011**	**-0.017**	**-0.004**	**0.007**
Pre – 5min	-0.003	-0.009	0.003	0.895
Pre – 15min	-0.003	-0.009	0.003	0.903
Pre – 90min	-0.002	-0.008	0.004	0.967
0min – 5min	0.008	0.002	0.014	0.104
0min – 15min	0.008	0.002	0.014	0.098
0min – 90min	0.009	0.002	0.015	0.054
5min – 15min	0.000	-0.006	0.006	1.000
5min – 90min	0.001	-0.005	0.007	0.999
15min – 90min	0.001	-0.006	0.007	0.999
	**ARA-S (ng/ml) (adjusted for CBD:THC ratio, visit number and time between visits)**			
**Pre – 0min**	**-0.011**	**-0.017**	**-0.004**	**0.008**
Pre – 5min	-0.003	-0.009	0.003	0.890
Pre – 15min	-0.002	-0.008	0.004	0.964
Pre – 90min	-0.002	-0.008	0.004	0.977
0min – 5min	0.008	0.001	0.014	0.114
0min – 15min	0.008	0.002	0.015	0.060
0min – 90min	0.009	0.002	0.015	0.050
5min – 15min	0.001	-0.005	0.007	0.999
5min – 90min	0.001	-0.005	0.007	0.998
15min – 90min	0.000	-0.006	0.006	1.000

eTable 3 continued.

Contrast	Estimated marginal mean difference	Lower 95% CI	Upper 95% CI	p-value
	**aLEA (ng/ml)**			
Pre – 0min	0.001	-0.003	0.005	0.995
Pre – 5min	0.002	-0.002	0.005	0.918
Pre – 15min	0.002	-0.002	0.006	0.843
Pre – 90min	0.002	-0.002	0.006	0.782
0min – 5min	0.001	-0.003	0.005	0.992
0min – 15min	0.001	-0.003	0.005	0.971
0min – 90min	0.001	-0.002	0.005	0.945
5min – 15min	0.000	-0.003	0.004	1.000
5min – 90min	0.001	-0.003	0.004	0.998
15min – 90min	0.000	-0.004	0.004	1.000
	**aLEA (ng/ml) (adjusted for CBD:THC ratio, visit number and time between visits)**			
Pre – 0min	0.001	-0.003	0.005	0.993
Pre – 5min	0.002	-0.002	0.005	0.921
Pre – 15min	0.002	-0.002	0.006	0.861
Pre – 90min	0.002	-0.002	0.006	0.779
0min – 5min	0.001	-0.003	0.005	0.995
0min – 15min	0.001	-0.003	0.005	0.981
0min – 90min	0.001	-0.002	0.005	0.952
5min – 15min	0.000	-0.004	0.004	1.000
5min – 90min	0.001	-0.003	0.004	0.998
15min – 90min	0.000	-0.004	0.004	1.000

eTable 3 continued.

Contrast	Estimated marginal mean difference	Lower 95% CI	Upper 95% CI	p-value
	**gLEA (ng/ml)**			
Pre – 0min	0.001	-0.002	0.004	0.891
Pre – 5min	0.002	-0.001	0.005	0.788
Pre – 15min	0.001	-0.002	0.004	0.940
Pre – 90min	0.000	-0.003	0.003	1.000
0min – 5min	0.000	-0.003	0.003	0.999
0min – 15min	0.000	-0.003	0.003	1.000
0min – 90min	-0.001	-0.004	0.002	0.920
5min – 15min	-0.001	-0.004	0.002	0.996
5min – 90min	-0.002	-0.005	0.001	0.831
15min – 90min	-0.001	-0.004	0.002	0.960
	**gLEA (ng/ml) (adjusted for CBD:THC ratio, visit number and time between visits)**			
Pre – 0min	0.001	-0.002	0.004	0.892
Pre – 5min	0.002	-0.001	0.005	0.784
Pre – 15min	0.001	-0.002	0.004	0.965
Pre – 90min	0.000	-0.003	0.003	1.000
0min – 5min	0.000	-0.003	0.003	0.999
0min – 15min	0.000	-0.003	0.003	0.999
0min – 90min	-0.001	-0.004	0.002	0.918
5min – 15min	-0.001	-0.004	0.002	0.989
5min – 90min	-0.002	-0.005	0.001	0.821
15min – 90min	-0.001	-0.004	0.002	0.977

eTable 4. Pre-inhalation plasma concentrations vs. visit number – Model 4a.

Statistically significant rows are presented in bold.

Contrast	Estimated marginal mean difference	Lower 95% CI	Upper 95% CI	p-value
	**Anandamide (ng/ml)**			
Visit 1 – Visit 2	0.006	-0.030	0.042	0.988
1 – 3	0.021	-0.015	0.057	0.651
**1 – 4**	**0.060**	**0.024**	**0.096**	**0.007**
2 – 3	0.015	-0.021	0.051	0.838
**2 – 4**	**0.054**	**0.018**	**0.090**	**0.019**
3 – 4	0.039	0.003	0.075	0.154
	**Anandamide (ng/ml) (adjusted for time between visits)**			
1 – 2	0.006	-0.035	0.048	0.990
1 – 3	0.025	-0.015	0.065	0.606
**1 – 4**	**0.063**	**0.024**	**0.103**	**0.010**
2 – 3	0.019	-0.018	0.055	0.745
**2 – 4**	**0.057**	**0.021**	**0.093**	**0.013**
3 – 4	0.038	0.002	0.074	0.156
	**2-AG (ng/ml)**			
1 – 2	0.713	-1.171	2.596	0.877
1 – 3	-0.927	-2.810	0.957	0.765
1 – 4	-0.771	-2.654	1.113	0.850
2 – 3	-1.639	-3.523	0.244	0.317
2 – 4	-1.483	-3.367	0.400	0.407
3 – 4	0.156	-1.727	2.040	0.998
	**2-AG (ng/ml) (adjusted for time between visits)**			
1 – 2	1.070	-0.990	3.130	0.734
1 – 3	-0.452	-2.447	1.543	0.970
1 – 4	-0.327	-2.301	1.646	0.988
2 – 3	-1.522	-3.358	0.314	0.360
2 – 4	-1.397	-3.237	0.443	0.439
3 – 4	0.125	-1.696	1.946	0.999

eTable 4 continued.

Contrast	Estimated marginal mean difference	Lower 95% CI	Upper 95% CI	p-value
	**Arachidonic acid (ng/ml)**			
Visit 1 – Visit 2	25.080	-30.462	80.623	0.808
1 – 3	5.628	-49.914	61.171	0.997
1 – 4	45.946	-9.596	101.489	0.362
2 – 3	-19.452	-74.995	36.090	0.900
2 – 4	20.866	-34.677	76.408	0.880
3 – 4	40.318	-15.225	95.860	0.480
	**Arachidonic acid (ng/ml) (adjusted for time between visits)**			
1 – 2	24.413	-38.909	87.735	0.871
1 – 3	8.438	-52.837	69.712	0.993
1 – 4	48.571	-12.031	109.174	0.391
2 – 3	-15.975	-72.233	40.283	0.943
2 – 4	24.158	-32.240	80.557	0.832
3 – 4	40.134	-15.653	95.920	0.487
	**DEA (ng/ml)**			
1 – 2	0.003	-0.005	0.011	0.866
1 – 3	0.004	-0.004	0.012	0.694
**1 – 4**	**0.011**	**0.003**	**0.019**	**0.031**
2 – 3	0.001	-0.007	0.009	0.989
2 – 4	0.008	0.000	0.016	0.192
3 – 4	0.007	-0.001	0.015	0.335
	**DEA (ng/ml) (adjusted for time between visits)**			
1 – 2	0.002	-0.008	0.011	0.988
1 – 3	0.004	-0.005	0.012	0.848
1 – 4	0.010	0.002	0.019	0.086
2 – 3	0.002	-0.006	0.010	0.954
2 – 4	0.009	0.001	0.017	0.130
3 – 4	0.007	-0.001	0.015	0.329

eTable 4 continued.

Contrast	Estimated marginal mean difference	Lower 95% CI	Upper 95% CI	p-value
	**OEA (ng/ml)**			
Visit 1 – Visit 2	-0.008	-0.205	0.189	1.000
1 – 3	0.029	-0.168	0.226	0.991
1 – 4	0.212	0.014	0.409	0.151
2 – 3	0.037	-0.160	0.234	0.982
2 – 4	0.220	0.022	0.417	0.128
3 – 4	0.183	-0.015	0.380	0.263
	**OEA (ng/ml) (adjusted for time between visits)**			
1 – 2	-0.083	-0.310	0.143	0.886
1 – 3	-0.036	-0.254	0.183	0.988
1 – 4	0.151	-0.065	0.368	0.512
2 – 3	0.048	-0.153	0.248	0.965
2 – 4	0.235	0.034	0.435	0.100
3 – 4	0.187	-0.012	0.385	0.249
	**SEA (ng/ml)**			
1 – 2	0.002	-0.256	0.260	1.000
1 – 3	-0.219	-0.477	0.039	0.339
1 – 4	0.094	-0.164	0.352	0.889
2 – 3	-0.222	-0.480	0.037	0.329
2 – 4	0.091	-0.167	0.349	0.897
3 – 4	0.313	0.055	0.571	0.082
	**SEA (ng/ml) (adjusted for time between visits)**			
1 – 2	-0.007	-0.290	0.276	1.000
1 – 3	-0.260	-0.534	0.015	0.246
1 – 4	0.056	-0.216	0.328	0.977
2 – 3	-0.253	-0.508	0.002	0.209
2 – 4	0.063	-0.193	0.319	0.962
3 – 4	0.316	0.062	0.569	0.070

eTable 4 continued.

Contrast	Estimated marginal mean difference	Lower 95% CI	Upper 95% CI	p-value
	**ARA-S (ng/ml)**			
Visit 1 – Visit 2	-0.002	-0.012	0.008	0.981
1 – 3	-0.005	-0.016	0.005	0.726
1 – 4	0.004	-0.006	0.015	0.840
2 – 3	-0.003	-0.014	0.007	0.913
2 – 4	0.006	-0.004	0.017	0.620
3 – 4	0.010	-0.001	0.020	0.246
	**ARA-S (ng/ml) (adjusted for time between visits)**			
1 – 2	-0.001	-0.013	0.011	0.998
1 – 3	-0.004	-0.015	0.007	0.902
1 – 4	0.006	-0.006	0.017	0.747
2 – 3	-0.003	-0.013	0.008	0.949
2 – 4	0.007	-0.004	0.017	0.570
3 – 4	0.010	-0.001	0.020	0.255
	**aLEA (ng/ml)**			
1 – 2	-0.005	-0.013	0.002	0.480
1 – 3	0.000	-0.007	0.007	1.000
1 – 4	0.002	-0.005	0.010	0.918
2 – 3	0.005	-0.002	0.013	0.494
2 – 4	0.008	0.000	0.015	0.165
3 – 4	0.002	-0.005	0.010	0.910
	**aLEA (ng/ml) (adjusted for time between visits)**			
1 – 2	-0.007	-0.015	0.001	0.314
1 – 3	-0.001	-0.009	0.007	0.987
1 – 4	0.001	-0.007	0.009	0.991
2 – 3	0.006	-0.002	0.013	0.403
2 – 4	0.008	0.001	0.016	0.118
3 – 4	0.003	-0.005	0.010	0.902

eTable 4 continued.

Contrast	Estimated marginal mean difference	Lower 95% CI	Upper 95% CI	p-value
	**gLEA (ng/ml)**			
Visit 1 – Visit 2	-0.004	-0.011	0.002	0.563
1 – 3	0.001	-0.006	0.008	0.993
1 – 4	0.000	-0.007	0.006	1.000
2 – 3	0.005	-0.001	0.012	0.395
2 – 4	0.004	-0.003	0.011	0.618
3 – 4	-0.001	-0.008	0.005	0.984
	**gLEA (ng/ml) (adjusted for time between visits)**			
1 – 2	-0.004	-0.012	0.003	0.625
1 – 3	0.001	-0.006	0.008	0.995
1 – 4	0.000	-0.007	0.007	1.000
2 – 3	0.005	-0.001	0.012	0.388
2 – 4	0.004	-0.003	0.011	0.609
3 – 4	-0.001	-0.008	0.005	0.984

eTable 5. Pre-inhalation plasma concentrations of AEA and DEA vs. total CBD dose from previous visits – Models 4b, 4c and 4d.

Possible previous doses of CBD at beginning of:
Visit 2: 0, 10, 20 or 30mg
Visit 3: 10, 20, 30, 40 or 50mg
Visit 4: 30, 40, 50 or 60mg

Contrast	Estimated marginal mean difference	Lower 95% CI	Upper 95% CI	p-value
	**Visit 2 - Anandamide (ng/ml) (adjusted for time between visits)**			
0mg – 10mg	0.007	-0.093	0.108	0.999
0mg – 20mg	0.028	-0.076	0.131	0.949
0mg – 30mg	0.044	-0.076	0.164	0.881
10mg – 20mg	0.020	-0.079	0.119	0.976
10mg – 30mg	0.036	-0.079	0.151	0.918
20mg – 30mg	0.016	-0.103	0.136	0.993
	**Visit 3 – Anandamide (ng/ml) (adjusted for time between visits)**			
30mg – 40mg	-0.020	-0.146	0.105	0.998
30mg – 50mg	0.004	-0.105	0.113	1.000
30mg – 60mg	0.018	-0.115	0.151	0.999
30mg – 70mg	0.086	-0.049	0.221	0.700
40mg – 50mg	0.024	-0.081	0.129	0.990
40mg – 60mg	0.038	-0.087	0.164	0.972
40mg – 70mg	0.106	-0.031	0.243	0.530
50mg – 60mg	0.014	-0.100	0.128	0.999
50mg – 70mg	0.082	-0.040	0.203	0.656
60mg – 70mg	0.068	-0.076	0.211	0.875
	**Visit 4 – Anandamide (ng/ml) (adjusted for time between visits)**			
30mg – 40mg	0.038	-0.080	0.053	0.972
30mg – 50mg	0.024	-0.070	0.054	0.990
30mg – 60mg	0.086	-0.075	0.053	0.700
40mg – 50mg	0.018	-0.066	0.078	0.999
40mg – 60mg	0.004	-0.070	0.076	1.000
50mg – 60mg	-0.020	-0.072	0.067	0.998

eTable 5 continued.

Contrast	Estimated marginal mean difference	Lower 95% CI	Upper 95% CI	p-value
	**Visit 2 - DEA (ng/ml) (adjusted for time between visits)**			
0mg – 10mg	0.014	-0.005	0.033	0.470
0mg – 20mg	0.017	-0.003	0.037	0.351
0mg – 30mg	-0.001	-0.024	0.022	1.000
10mg – 20mg	0.003	-0.017	0.022	0.993
10mg – 30mg	-0.015	-0.037	0.007	0.525
20mg – 30mg	-0.018	-0.041	0.005	0.422
	**Visit 3 – DEA (ng/ml) (adjusted for time between visits)**			
30mg – 40mg	-0.003	-0.034	0.028	1.000
30mg – 50mg	0.000	-0.027	0.026	1.000
30mg – 60mg	0.013	-0.019	0.044	0.931
30mg – 70mg	0.022	-0.010	0.055	0.634
40mg – 50mg	0.003	-0.024	0.029	1.000
40mg – 60mg	0.016	-0.015	0.047	0.844
40mg – 70mg	0.025	-0.008	0.059	0.549
50mg – 60mg	0.013	-0.014	0.040	0.871
50mg – 70mg	0.023	-0.006	0.052	0.517
60mg – 70mg	0.010	-0.025	0.044	0.978
	**Visit 4 – DEA (ng/ml) (adjusted for time between visits)**			
30mg – 40mg	-0.001	-0.015	0.014	1.000
30mg – 50mg	-0.002	-0.016	0.012	0.994
30mg – 60mg	0.007	-0.007	0.021	0.767
40mg – 50mg	-0.001	-0.017	0.015	0.998
40mg – 60mg	0.007	-0.009	0.024	0.798
50mg – 60mg	0.009	-0.007	0.024	0.675

eResults

Sex differences

There were no significant sex differences between the endocannabinoids or related non-cannabinoid lipid responses to THC or CBD, with the exception of Model 3b (effect of drug administration: all CBD:THC ratios) and Model 4a (effect of visit number on plasma concentrations) for SEA:

Overall effect of THC

Mean SEA concentration in men rose by 127.2% (0.220ng/ml [95%CI: 0.075–0.364], $t(854)=2.981$, $p=0.025$) immediately post-inhalation, before falling to pre-inhalation levels by 5min. In women, there was no significant change in SEA concentration across the sampling timepoints ($p>0.05$). However, an outlier was identified using Rosner's generalised extreme Studentised deviate test in a male participant (0 min, value= 4.527ng/ml, 55.8% higher than next highest value for that timepoint). When this outlier value was removed, there was no longer a significant change in mean SEA concentration in men ($p=0.060$).

Effect of visit order on endocannabinoid levels

Over the four experimental visits, pre-inhalation plasma levels of SEA did not significantly change in men ($p>0.05$) but did alter in women. Between visits 1 and 3, mean SEA concentrations rose by 315.6% (0.584ng/ml [95%CI: 0.192–0.975], $t(140)=2.949$, $p=0.019$), before falling again by visit 4 (-76.3%, 0.594ng/ml [95%CI: 0.221–0.967], $t(131)=3.153$, $p=0.011$). However, an outlier was identified using Rosner's generalised extreme Studentised deviate test in a female participant (visit 3, value= 5.830ng/ml, 115.6% higher than next highest value for that visit). When this outlier value was removed, there was no longer a significant change in pre-inhalation SEA between visits in women ($p\geq0.128$).

Effect of peak THC concentration

There was a negative correlation between peak THC plasma concentration and change in DEA plasma concentration immediately post-inhalation (R= -0.162 [95%CI: -0.018 to -0.300], $t(182)= -2.2181$, $p=0.028$). This was no longer significant, however, when the model was fully adjusted for pre-inhalation concentration of DEA ($p=0.279$).

There was no correlation between peak THC concentration and change in any of the other analytes ($p>0.05$).

References

1. Selzer ML. The Michigan Alcoholism Screening Test: The Quest for a New Diagnostic Instrument. Am J Psychiatry 1971;127(12):1653–1658; doi: 10.1176/ajp.127.12.1653.
2. Heatherton T, Kozlowski L, Frecker R, et al. The Fagerstrom Test for Nicotine Dependence: A Revision of the Fagerstrom Tolerance Questionnaire. Br J Addict 1991;86(9):1119–1127.
3. Skinner HA. The Drug Abuse Screening Test. Int J 1983;I:363–371.
4. Stefanis NC, Hanssen M, Smirnis NK, et al. Evidence That Three Dimensions of Psychosis Have a Distribution in the General Population. Psychol Med 2002;32(2):347–58.
5. Brandt J. The Hopkins Verbal Learning Test: Development of a New Memory Test with Six Equivalent Forms. Clin Neuropsychol 1991;5(2):125–142; doi: 10.1080/13854049108403297.
6. Kay SR, Fiszbein A and Opler LA. The Positive and Negative Syndrome Scale (PANSS) for Schizophrenia. Schizophr Bull 1987;13(2):261–76.
7. Mason OJ, Morgan CJM, Stefanovic A, et al. The Psychotomimetic States Inventory (PSI): Measuring Psychotic-Type Experiences from Ketamine and Cannabis. Schizophr Res 2008;103(1–3):138–142; doi: 10.1016/j.schres.2008.02.020.
8. Freeman TP, Morgan CJA, Vaughn-Jones J, et al. Cognitive and Subjective Effects of Mephedrone and Factors Influencing Use of a ‘New Legal High.’ Addiction 2012;107(4):792–800; doi: 10.1111/j.1360-0443.2011.03719.x.
9. Freeman D, Pugh K, Green C, et al. A Measure of State Persecutory Ideation for Experimental Studies. J Nerv Ment Dis 2007;195(9):781–784; doi: 10.1097/NMD.0b013e318145a0a9.
10. Huestis MA. Pharmacokinetics and Metabolism of the Plant Cannabinoids, Δ9-Tetrahydrocannibinol, Cannabidiol and Cannabinol. In: Handbook of Experimental Pharmacology. (Pertwee RG. ed) Springer Nature: Berlin; 2005; pp. 657–690; doi: 10.1007/3-540-26573-2-23.
11. Hartman RL, Brown TL, Milavetz G, et al. Controlled Cannabis Vaporizer Administration: Blood and Plasma Cannabinoids with and without Alcohol. Clin Chem 2015;61(6):850–869; doi: 10.1373/clinchem.2015.238287.
